# Into the Blue: Ketene Multicomponent Reactions under Visible Light

**DOI:** 10.1021/acs.joc.1c00278

**Published:** 2021-04-06

**Authors:** Pietro Capurro, Chiara Lambruschini, Paola Lova, Lisa Moni, Andrea Basso

**Affiliations:** Dipartimento di Chimica e Chimica Industriale, Università degli Studi di Genova, Via Dodecaneso 31, 16146 Genova, Italy

## Abstract

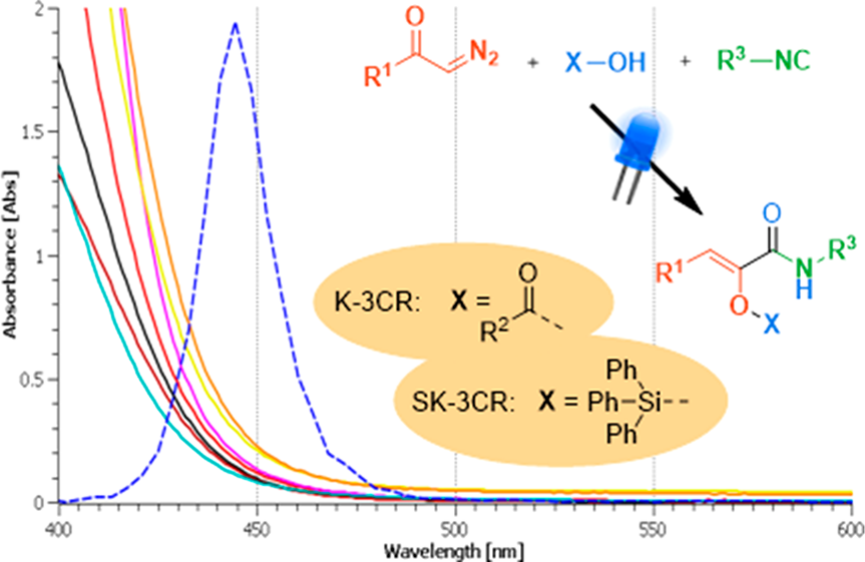

For the first time, a detailed study on the photophysical properties of variously substituted diazoketones and on their photoreactivity under blue LED irradiation was carried out. Despite very limited absorbance in the visible region, we have demonstrated that, independently from their structure, α-diazoketones all undergo a very efficient Wolff rearrangement. Contrarily to the same UV-mediated reaction, where photons can give rise to side processes, in this case, almost all absorbed photons are selective and effective, and the quantum yield is close to 100%. If the rearrangement is carried out in the presence of isocyanides and carboxylic acids/silanols, the photoreactivity is not affected, and the resulting ketenes can afford α-acyloxy- and α-silyloxyacrylamides through two distinct multicomponent reactions, performed both in batch and under continuous flow, with improved selectivity and broader scope. These photoinduced multicomponent reactions can be coupled with other visible-light-mediated transformations, thus increasing the diversity of the molecules obtainable by this approach.

## Introduction

Diazocompounds are versatile substrates, able to easily undergo either carbene generation or Wolff rearrangement to ketenes, which subsequently undergo various transformations. Modern chemistry of diazo compounds is dominated by transition metal catalysis, although photoinduced and thermal decompositions have also found application.^1^ Photoinitiated reactions have experienced a revival of interest due to the increased understanding of photochemical mechanisms. The photoinitiated decomposition of diazocompounds is mainly induced by UV irradiation, and only recently the use of visible light has been reported by some authors. For example, Konopelski assembled enantiomerically pure β-lactams from α-diazo-*N*-methoxy-*N*-methyl β-ketoamides, exploiting a UV or CFL-induced Wolff rearrangement,^2^ and Burtoloso reported the use of white LED in the Arndt–Eistert homologation of simple aliphatic and aromatic diazoketones.^3^ In these cases, however, the emission spectra of the light sources were not correlated with the absorbance spectra of the substrates. On the other hand, Lu and Xiao used blue LED activation on various aryl methyl diazoketones,^4,5^ demonstrating that these compounds had a discrete absorption in the 410–500 nm region. Phenyl methyl diazoketone was also employed by Song, who monitored the Wolff rearrangement under blue LED irradiation by analyzing the ^1^H NMR spectra of the reaction mixture, reporting almost quantitative conversion after 10 h.^6^ However, the author reported that simple monosubstituted aromatic α-diazoketones and aliphatic α-diazoketones were unreactive substrates. Zhou reported blue-light-promoted cross-coupling of aryldiazoacetates and diazocarbonyl compounds, in which only the first was selectively activated by the absorption of light in the visible region, while the latter diazo compound remained inactive. This was justified by the authors by the “lack of appreciable absorbance” of diazocarbonyl compounds (including phenyldiazoketone) in the visible region.^7^

**Scheme 1:**
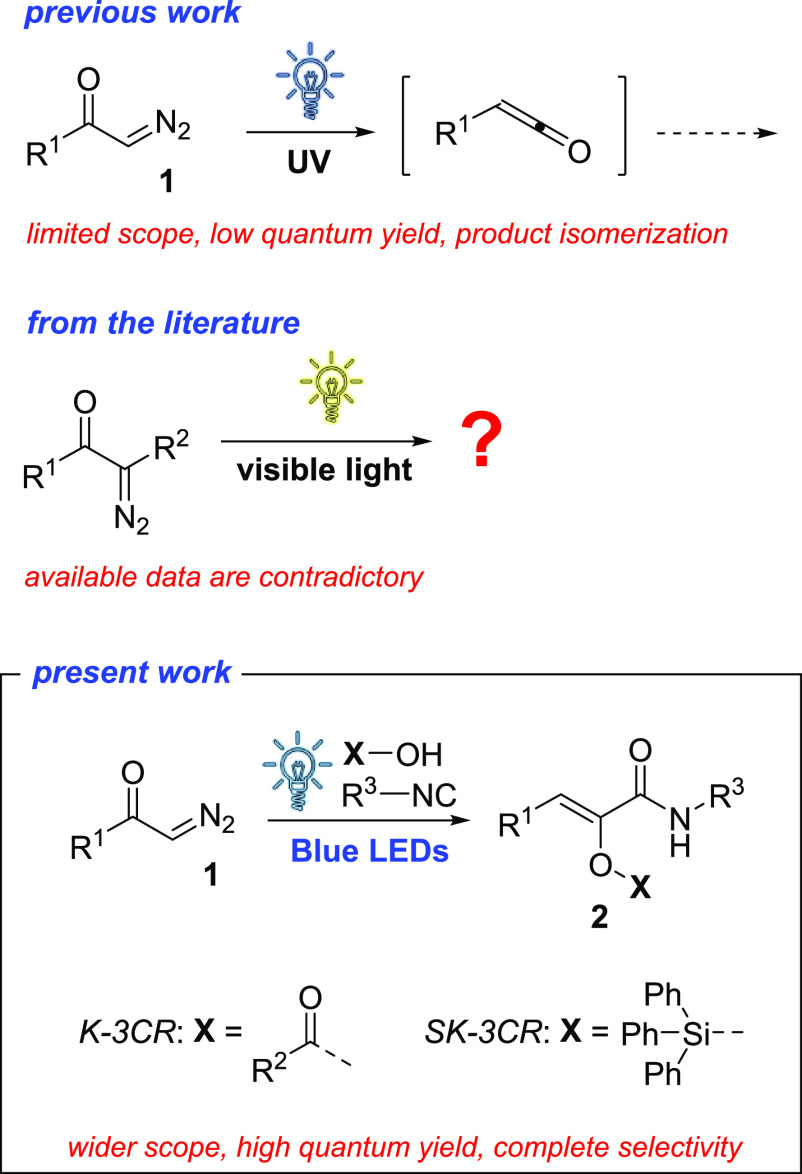
State of the Art of the Photoinduced Wolff Rearrangement of Diazocompounds and General Scheme for the Ketene Three-Component Reaction (K-3CR) and Sylilative Ketene Three-Component Reaction (SK-3CR) Reported in the Present Work

These data apparently contradict each other, and a rigorous study on the absorption spectra of diazoketones and on how these are correlated to their reactivity is lacking. In our personal experience, monosubstituted diazoketones **1** display a UV–vis absorbance spectrum with a maximum at about 250–300 nm and a negligible absorbance above 400 nm. For this reason, the ketene three-component reaction (K-3CR)^8^ and the silylative ketene three-component reaction (SK-3CR),^9^ recently developed in our laboratories, were successfully performed under UV irradiation at 254–360 nm (Scheme 1). These methods, however, had some drawbacks: to prevent that the same UV radiation-induced double-bond isomerization of the olefinic products **2**, the use of additives^10^ or, more recently, the employment of flow conditions,^11^ had to be introduced. Moreover, competitive absorbance of some substrates, like those possessing a nitrophenyl or an indole moiety, prevented the Wolff rearrangement from occurring, thus resulting in the failure of the multicomponent reaction.

## Results and Discussion

### Spectroscopic Studies

**Figure 1:**
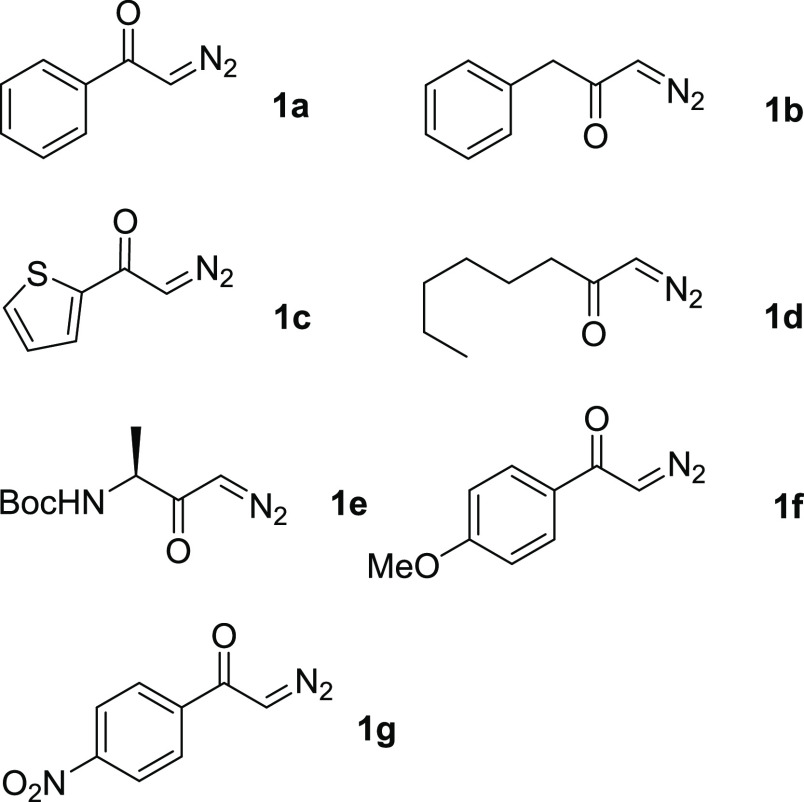
List of diazoketones employed in this study.

**Figure 2:**
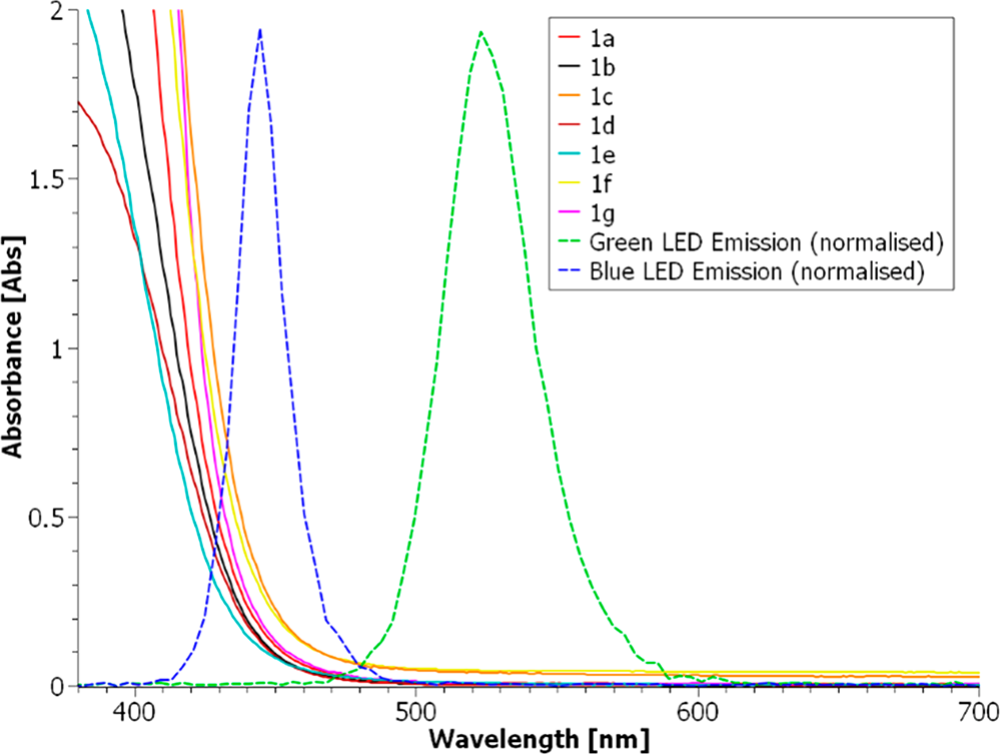
Absorption spectra for diazoketones **1a**–**g** (0.1 M in dichloromethane) in the region 380–700 nm and overlap with emission spectrum of blue and green LED.

Intrigued by the possibility to perform these multicomponent reactions under less energetic irradiation, puzzled by the contradictory literature, and aware of the fact that diazoketones are usually bright yellow solids, we decided to investigate the possibility to use blue LEDs instead of UV light to induce the Wolff rearrangement. We selected and synthesized seven different monosubstituted diazoketones (Figure 1), both aliphatic and aromatic, with EW and ED substituents, and determined the UV–vis spectra together with the molar extinction coefficient at 450 nm (Table 1 and Figure 2). This latter value was much smaller (in the range of 1–2 L mol^–1^ cm^–1^) than the one determined at the λ of maximum absorbance but was not negligible.

**Table 1:** Maximum Absorption and Molar Extinction Coefficient for Diazoketones **1a**–**g**

diazoketone	λ_max_ (nm)	ε_max_^a^^,^^b^	ε_450_^a^^,^^c^
**1a**	253	9600	1.21
**1b**	252	7600	0.96
**1c**	307	13500	2.29
**1d**	248	8300	0.90
**1e**	250	5200	0.84
**1f**	301	18400	2.11
**1g**	268	11100	1.34

a Expressed in L mol^–1^ cm^–1^.

b Determined in dichloromethane at 0.1–0.05 mM concentration.

c Determined in dichloromethane at 0.1 M concentration.

### K-3CR and Quantum Yield Determination

**Figure 3:**
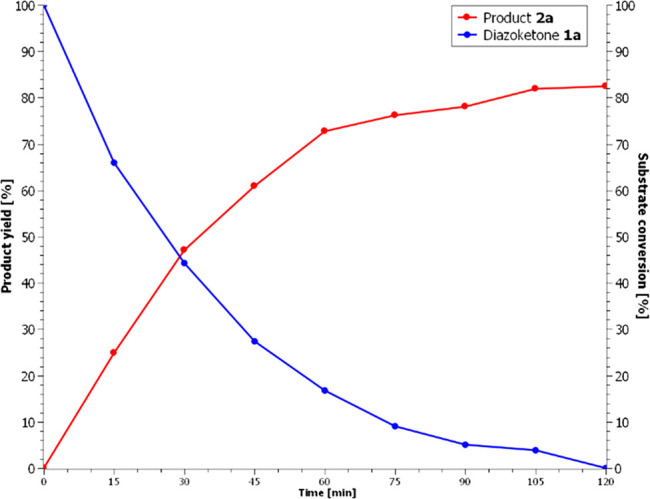
NMR study of the model reaction.

Considering these results, a solution of diazoketone **1a** in dichloromethane was irradiated at 450 nm with a high-power (1 W of radiant power) blue LED, and gratifyingly, complete consumption was observed in a few hours. It is worth noting that no consumption was observed when low-power blue LED stripes or high-power green LEDs were used instead. This could in part explain the contradictory results found in the literature. We then moved to test a model K-3CR, exposing a mixture of diazoketone **1a**, benzoic acid, and cyclohexyl isocyanide, dissolved in CDCl_3_, to irradiation with blue LED and monitoring the reaction by NMR. Then, 0.05 mmol of DMSO was added as an internal standard for the integration reference. Aliquots (100 μL) of the solution were sampled, diluted in an NMR tube to a total volume of 700 μL of CDCl_3_, and analyzed via ^1^H NMR at defined time intervals. To our delight, the reaction proceeded with the desired formation of product **2a**, concomitantly with the consumption of substrate **1a**, as illustrated in Figure 3. As expected, no isomerization of the double bond of acrylamide **2a** was observed, as this was found to occur only under UV irradiation. The reaction was performed and monitored twice: the first run was sampled every 15 min up to the complete disappearance of diazoketone **1a** (120 min); the second run was sampled every 5 min for 30 min. Integration of selected, noninterfered signals with respect to that of the internal standard (DMSO) allowed us to calculate the rate of disappearance of the starting reagents (**1a**, benzoic acid, and cyclohexyl isocyanide) and the rate of formation of product **2a**. The quantum yields for the Wolff rearrangement (disappearance of the diazoketone **1a**) and for the multicomponent step (formation of **2a**) were determined by measurement, in parallel, of the absorbed photons employing a fiber-based optical setup built *ad hoc*. The photon flow from the LED system was collected using an integrating sphere opportunely placed around the reaction vial and connected to a CMOS spectrometer (with a resolution of 1.4 nm) with an optical fiber. The estimation of the emitted number of photons was allowed by a precise calibration of the setup response using a calibrated white light source (see Supporting Information for additional details). From the results reported in Table 2, the quantum yield for the formation of product **2a** remains constant in the first 30 min of analysis, with an average value of 64%. The quantum yield for the disappearance of **1a**, on the other hand, decreases over time, with a starting value of 100% after 5 min and an average value of 89% over the first 30 min. This can be explained with the accumulation, in the reaction mixture, of absorbing impurities deriving from phenylketene, which subtract a small portion of photons to **1a**. The quantum yield for the formation of **2a**, on the other hand, is not affected by these impurities, as the interaction of the ketene with the other two reagents is the rate-limiting step of the process; thus, there is always an excess of ketene in the solution. The diazoketone disappearance quantum yield was found to be much higher than the one determined with UV (Wood’s lamp) irradiation, namely, 28%.^11^ These results demonstrate that, despite a very low molar extinction coefficient, the absorbed photons almost exclusively promote the Wolff rearrangement, resulting in a greatly improved efficiency of the present methodology.

**Table 2:** Disappearance Rates of Diazoketone **1a** and Formation Rate of the Product **2a** for the Model Reaction, Number of Photons Absorbed by the Reacting Species **1a**, and Quantum Yields (φ^–^, φ^K3CR^) for the Wolff Rearrangement and the K-3CR Multicomponent Process, Respectively

time [min]	**1a**^a^ [%]	**2a**^a^ [%]	*N*_photons_^b^	φ^–^ c	φ^K3CR^ d
0	100.0	0.0	1.24 × 10^18^		
5	84.8	9.1	2.60 × 10^19^	1.05	0.63
10	74.3	18.2	5.00 × 10^19^	0.93	0.66
15	64.8	26.5	7.29 × 10^19^	0.87	0.66
20	55.8	34.0	9.47 × 10^19^	0.84	0.65
25	46.7	40.0	1.15 × 10^20^	0.83	0.63
30	39.7	45.6	1.35 × 10^20^	0.80	0.61
average values				**0.89**	**0.64**

a Determined by integration of the NMR spectra.

b Calculated number of total photons absorbed.

c Quantum yield for the Wolff rearrangement (value calculated at each time interval).

d Quantum yield for the model K-3CR (value calculated at each time interval).

### K-3CR and SK-3CR in Batch and Flow

**Scheme 2:**
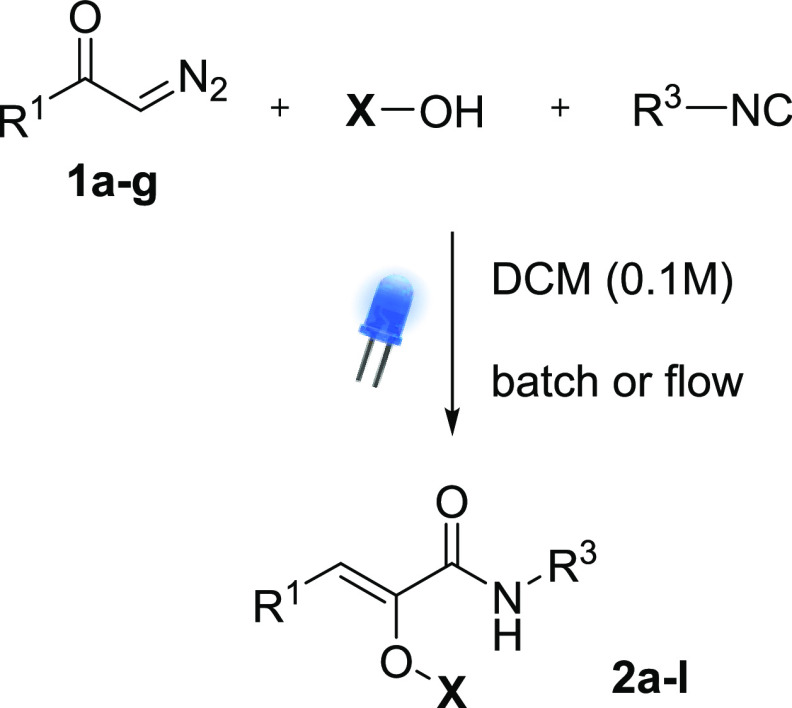
K-3CR and SK-3CR with Diazoketones **1a**–**g**

**Figure 4:**
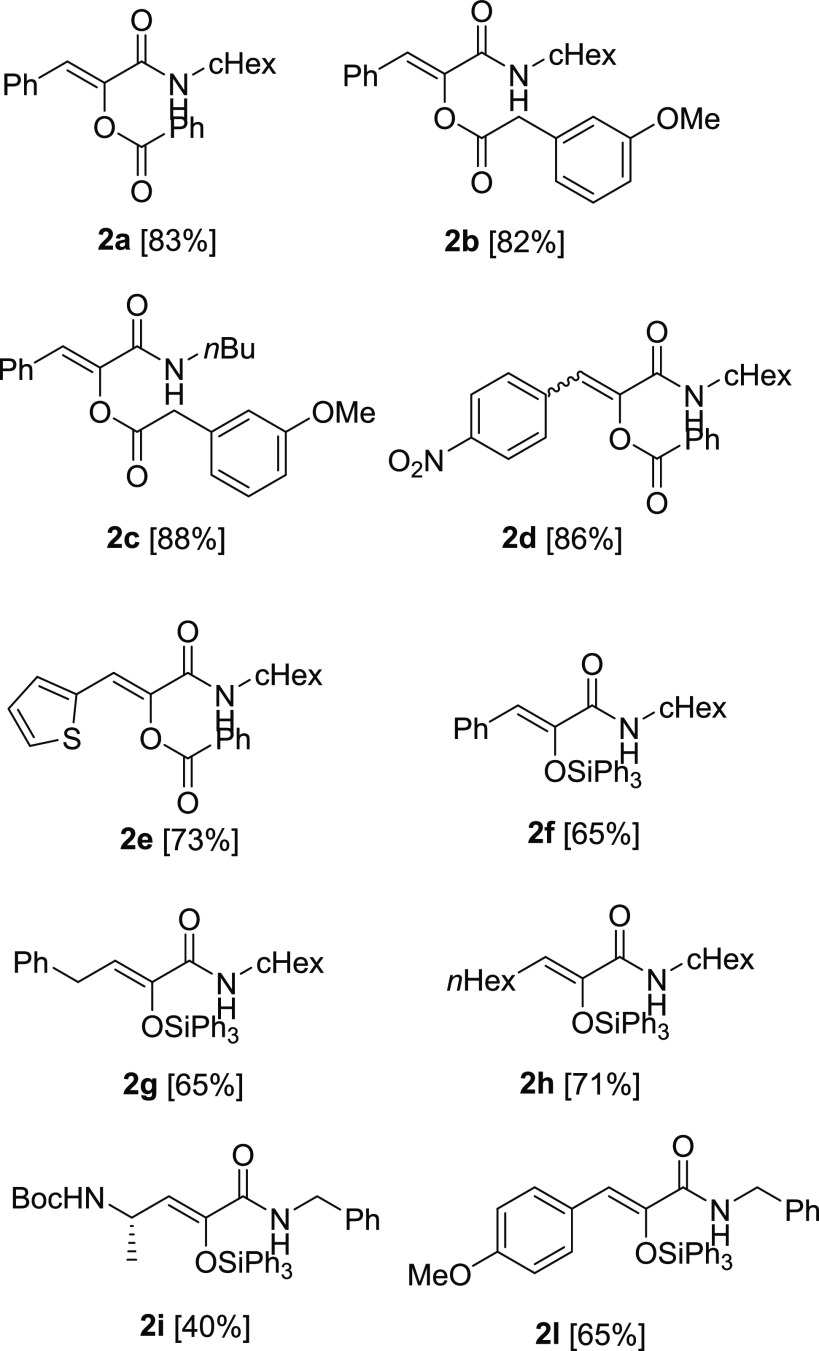
K-3CR and SK-3CR products with diazoketones **1a**–**g**. The given yield is that of the isolated product after column chromatography.

A series of K-3CR and SK-3CR was then performed, employing different combinations of diazoketones, isocyanides, and carboxylic acids/triphenylsilanol (Scheme 2). Test experiments, reported in the Supporting Information, excluded a change in the absorption profile around 450 nm when moving from pure diazoketone to the multicomponent mixtures. The results are shown in Figure 4. As expected from the absorption spectra (Figure 2), all of the diazoketones employed in this study gave the Wolff rearrangement with comparable reactivity, independently from their structure. This remarkable result is in contrast with what was previously reported.^6^

The advantages of the present methodology, in comparison with the UV-induced multicomponent reactions, are reported in Table 3. Clearly, E/Z selectivity dramatically improved by using a less energetic radiation. In fact, beside an overall yield that remained substantially unchanged, double bond isomerization was completely suppressed in most cases. The only exception is compound **2d** obtained by the reaction of diazoketone **1g** with benzoic acid and cyclohexyl isocyanide. In this case, however, another advantage is evident: the bathochromic effect of the nitro group in diazoketone **1g** prevented the UV-mediated Wolff rearrangement from occuring, as the absorbance of the aromatic ring overlapped the one of the diazoketone moiety. The use of blue LED, instead, allowed the K-3CR to take place cleanly with compound **2d** isolated in 86% yield. The poor E/Z selectivity, in this case, was the result of the same bathochromic effect on the olefinic product, with the ability to cause double bond isomerization even under visible light.

**Table 3:** Comparison of K-3CR and SK-3CR Performed with Blue LED and under UV Irradiation (without Additives) in Terms of Yield and E/Z Selectivity^a^

	batch conditions	
product	blue LED	UV (300 nm)
**2b**	82% (100:0)	65% (71:29)
**2c**	88% (100:0)	56% (66:34)
**2d**	86% (57:43)	
**2e**	73% (100:0)	37% (56:44)
	flow conditions	
**2l**	65% (100:0)	50% (67:33)
**2b**	77% (100:0)	46% (97:3)
**2g**	68% (100:0)	
**2l**	43% (100:0)	

a For UV reactions, yields are referred to as the major isomer.

The multicomponent reactions could be performed both in batch and under continuous flow conditions, and in this latter case, another advantage resulted from the possibility to perform in flow not only the K-3CR but, for the first time, also the SK-3CR, as this was unsuccessful employing UV radiation. The continuous flow apparatus was built in-house with the same high-power blue LEDs, and full details are given in the Supporting Information.

### Further Visible Light Transformations

**Scheme 3:**
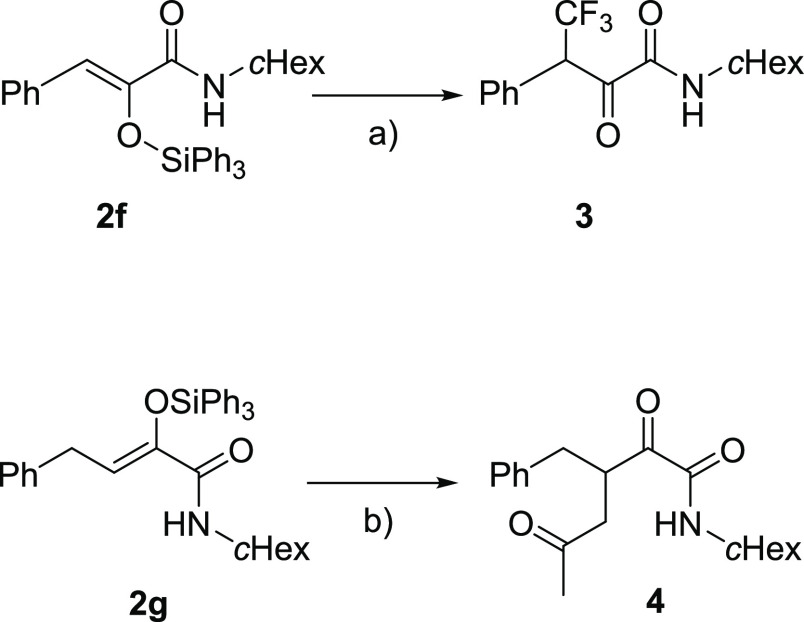
Coupling SK-3CR with Other Visible-Light-Mediated Processes Reagents and conditions: (a) Umemoto II (2 equiv), [Ru(bpy)_3_]Cl_2_ (1 mol %), MeCN (0.01 M), blue LED, flow 1.5 mL h^–1^, 32%; (b) 4-methoxyphenyl diazonium tetrafluoroborate (1 equiv), [Ru(bpy)_3_]Cl_2_ (1 mol %), acetone (0.01 M), blue LED, flow 3 mL h^–1^, 75%.

α-Substituted acrylamides **2** have been demonstrated to be valuable synthons for the synthesis of heterocycles^12^ and natural products.^13^ Having established this new methodology, we then moved to explore the possibility to couple the multicomponent reactions with visible-light-mediated functionalization of the resulting products. The first reaction we explored was a trifluoromethylation, which was first attempted on substrate **2f** with trifluoromethylsulfonyl chloride in the presence of eosin Y under green LED irradiation, unsuccessfully; eventually, the synthesis of **3** was achieved using Umemoto II reagent and Ru photoredox catalysis with blue light, both in batch and under continuous flow conditions (Scheme 3, top).^14^ In the second set of experiments, we coupled the SK-3CR with a photoredox generation of an acetonyl radical.^15^ The reaction was performed on the crude mixture derived from the synthesis of **2g** and was performed under continuous flow conditions, affording 1,4-diketone **4** in an overall 40% yield, based on two steps (Scheme 3, bottom). Although this transformation was performed as a separate step, the use of the crude mixture of the SK-3CR and of a flow system demonstrates, in principle, that, with the proper equipment, this synthetic route can be performed within the same reactor in one operation.

## Conclusion

In conclusion, a rigorous study on the absorption properties of 7 structurally different diazoketones has demonstrated that the Wolff rearrangement can occur under blue LED irradiation, independently of their structure, and that the process is more efficient than the one performed under UV irradiation, due to the higher selectivity of the less energetic photons. The Wolff rearrangement has been exploited in two distinct multicomponent reactions, namely, K-3CR and SK-3CR, affording the desired products with complete E/Z selectivity and even in those cases where the UV mediated process failed. The reactions are complete in a few hours, and quantum yields are remarkably high. We are currently applying the present approach in other transformations involving ketenes, in particular, on [2+2] cycloadditions, and we will report the results in due course.

## Experimental Section

### General Information

NMR spectra were recorded at 300 MHz (^1^H), 75 MHz (^13^C), and 282 MHz (^19^F), and the chemical shifts (δ) are expressed in parts per million relatively to tetramethylsilane (TMS) as an internal standard (0.00 ppm). Coupling constants are reported in hertz. NMR acquisitions were performed at 300 K, and CDCl_3_ was used as a solvent. HR-MS analyses were carried out on a Synapt G2 QToF mass spectrometer. MS signals were acquired from 50 to 1200 *m*/*z* in ESI positive ionization mode. UV–vis analyses were performed on a Varian Cary 50 Scan (190–1100 nm) using quartz cuvettes (Hellma Standard absorption cuvettes in Suprasil quartz) using nonanhydrous dichloromethane (cutoff ca. 230 nm) as a solvent, purchased from Sigma-Aldrich, and used without further purification. Reactions were monitored by TLC. TLC analyses were carried out on silica gel plates (thickness = 0.25 mm), viewed at UV (λ = 254 nm) and developed with Hanessian stain (dipping into a solution of (NH_4_)_4_MoO_4_·4H_2_O (21 g) and Ce(SO_4_)_2_·4H_2_O (1 g) in H_2_SO_4_ (31 mL) and H_2_O (469 mL) and warming). Column chromatography was performed with the “flash” methodology alternatively using 220–400 mesh silica, grade I alumina, or 60–100 mesh Florisil. Solvents employed as eluents and for all other routinary operations, as well as anhydrous solvents and all reagents used, were purchased from commercial suppliers and employed without any further purification.

Diazoketones **1a**–**g** were synthesized according to literature procedures,^16^ and analytical data were in accordance with the literature.^16,17^

### General Procedure for K-3CR and SK-3CR in Batch

The desired diazoketone (0.3 mmol, 1 equiv, 0.1 M) and the acidic component (silanol or carboxylic acid, 0.3 mmol, 1 equiv, 0.1 M) were added to a vial. The vial was sealed and degassed with argon for 5 min. The solvent (dry DCM, 3 mL) and the isocyanide (0.3 mmol, 1 equiv, 0.1 M) were added; then, the solution was degassed for 5 min with argon. The mixture was then irradiated at a constant temperature (below 20 °C) at 450 nm (blue LEDs) under magnetic stirring until complete consumption of the diazoketone (2 h to overnight) upon TLC analysis (PE/EtOAc 4:1, UV/Hanessian stain). Purification: products **2a**–**e**, **2g**–**i**, flash chromatography (SiO_2_, PE/EtOAc); products **2f** and **2l**, precipitation from 2 mL of Et_2_O (−24 °C overnight).

### General Procedure for K-3CR and SK-3CR in Flow

Prior to irradiation, the flow apparatus was flushed with nitrogen and conditioned with dry DCM (including the airtight syringe). The solution of reactants (see batch conditions above) was loaded into the loading loop and then eluted at 1 mL h^–1^ (residence time 3 h) for a total of 12 h to ensure complete collection of the irradiated solution. The products were then purified as previously described for the batch conditions.

#### (*Z*)-3-(Cyclohexylamino)-3-oxo-1-phenylprop-1-en-2-yl benzoate **2a**

White solid, mp 163.7–165.2 °C. *R_f_* = 0.34 (DCM/EtOAc/PE 1:1:5.5). Yield: 83% (87 mg). ^1^H NMR: δ 8.19 [dd, *J* = 8.4, 1.5, 2H], 7.68 [tt, *J* = 7.5, 1.2, 1H], 7.56–7.50 [m, 4H], 7.33 [s, 1H], 7.28–7.24 [m, 3H], 6.03 [d br, *J* = 8.0, 1H], 3.94–3.81 [m, 1H], 1.97–1.09 [m, 10H]. ^13^C{^1^H} NMR: δ 163.7, 161.7, 140.0, 134.3, 132.5, 130.3, 129.6, 129.1, 129.0, 128.7, 128.4, 123.6, 48.6, 32.9, 25.5, 24.8. HRMS (ESI) *m*/*z*: [M + H]^+^ calcd for C_22_H_24_NO_3_, 350.1756; found, 350.1757.

#### (*Z*)-3-(Cyclohexylamino)-3-oxo-1-phenylprop-1-en-2-yl 2-(3-methoxyphenyl)acetate **2b**

White solid, mp 89.0–91.2 °C. *R_f_* = 0.27 (DCM/EtOAc/PE 1:1:5.5). Yield: 82% (97 mg). ^1^H NMR: δ 7.43–7.25 [m, 7H], 7.00–6.89 [m, 3H], 5.46 [d br, *J* = 8.4, 1H], 3.80 [s, 3H], 3.79 [s, 2H], 3.76–3.65 [m,1H], 1.77–0.73 [m, 10H]. ^13^C{^1^H} NMR: δ 167.7, 161.2, 160.2, 139.4, 134.2, 132.5, 130.4, 129.6, 129.2, 128.7, 124.0, 121.7, 115.0, 113.4, 55.3, 48.2, 41.9, 32.7, 25.5, 24.9. HRMS (ESI) *m*/*z*: [M + H]^+^ calcd for C_24_H_28_NO_4_, 394.2018; found, 394.2012.

#### (*Z*)-3-(Butylamino)-3-oxo-1-phenylprop-1-en-2-yl 2-(3-methoxyphenyl)acetate **2c**

Yellow oil. *R_f_* = 0.27 (EtOAc/PE 3:7). Yield: 88% (97 mg). ^1^H NMR: δ 7.43–7.29 [m, 7H], 7.00–6.89 [m, 3H], 5.52 [t br, *J* = 5.1, 1H], 3.81 [m, 5H], 3.13 [q, *J* = 6.9, 2H], 1.33–1.14 [m, 4H], 0.88 [t, *J* = 6.9, 3H]. ^13^C{^1^H} NMR: δ 167.8, 162.3, 160.3, 139.4, 134.2, 132.5, 130.4, 129.6, 129.3, 128.8, 124.2, 121.7, 115.0, 113.6, 55.4, 42.0, 39.6, 31.5, 20.1, 13.9. HRMS (ESI) *m*/*z*: [M + H]^+^ calcd for C_22_H_26_NO_4_, 368.1862; found, 368.1852.

#### (*E*)-3-(Cyclohexylamino)-3-oxo-1-(4-nitrophenyl)prop-1-en-2-yl benzoate (*E*)-**2d**

Yellow foam. *R_f_* = 0.61 (EtOAc/PE 1:3). Yield: 49% (58 mg). ^1^H NMR: δ 8.25–8.19 [m, 2H], 8.17–8.10 [m, 2H], 7.73–7.58 [m, 3H], 7.52 [t, *J* = 7.8 Hz, 2H], 6.74 [s, 1H], 5.82 [d, *J* = 8.3 Hz, 1H], 3.81 [m, 1H], 1.91–1.77 [m, 2H], 1.68–1.56 [m, 2H], 1.43–0.94 [m, 6H]. ^13^C{^1^H} NMR: δ 164.9, 160.9, 147.4, 144.6, 139.1, 134.3, 130.3, 130.0, 128.8, 128.1, 123.6, 121.6, 48.5, 32.4, 25.3, 24.6.HRMS (ESI) *m*/*z*: [M + H]^+^ calcd for C_22_H_22_N_2_O_5_, 394.1529; found, 394.1536.

#### (*Z*)-3-(Cyclohexylamino)-3-oxo-1-(4-nitrophenyl)prop-1-en-2-yl benzoate (*Z*)-**2d**

Yellow foam. *R*_*f*_ = 0.51 (EtOAc/PE 1:3). Yield: 37% (44 mg). ^1^H NMR: δ 8.21–8.10 [m, 4H], 7.78–7.70 [m, 1H], 7.66 [dd, *J* = 9.0, 0.5 Hz, 2H], 7.61–7.53 [m, 2H], 7.39 [s, 1H], 5.93 [d, *J* = 8.2 Hz, 1H], 3.97–3.79 [m, 1H], 1.96 [dd, *J* = 12.2, 3.7 Hz, 2H], 1.75–1.62 [m, 2H], 1.47–1.07 [m, 6H]. ^13^C{^1^H} NMR: δ 163.2, 160.8, 147.4, 142.6, 139.0, 134.8, 130.3, 130.0, 129.2, 127.7, 123.9, 121.3, 48.8, 32.8, 25.4, 24.7. HRMS (ESI) *m*/*z*: [M + H]^+^ calcd for C_22_H_22_N_2_O_5_, 394.1529; found, 394.1523.

#### (*Z*)-3-(Cyclohexylamino)-3-oxo-1-(thiophen-2-yl)prop-1-en-2-yl benzoate **2e**

Brown solid, mp 129.5–131.5 °C. *R_f_* = 0.32 (DCM/EtOAc/PE 2:1:7). Yield: 73% (78 mg). ^1^H NMR: δ 8.29 [dd, *J* = 8.4, 1.5, 2H], 7.72 [tt, *J* = 7.5, 1.5, 1H], 7.64 [s, 1H], 7.58 [tt, *J* = 7.8, 1.5, 2H], 7.33 [dt, *J* = 5.1, 1.2, 1H], 7.28–7.27 [m, 1H], 7.02 [dd, *J* = 5.1, 3.7, 1H], 5.80 [d br, *J* = 7.8, 1H], 3.95–3.82 [m, 1H], 1.97–1.07 [m, 10H]. ^13^C{^1^H} NMR: δ 163.9, 161.3, 137.9, 134.9, 134.4, 131.7, 130.7, 129.5, 129.0, 128.6, 127.2, 118.2, 48.7, 33.0, 25.6, 24.8. HRMS (ESI) *m*/*z*: [M + H]^+^ calcd for C_20_H_22_NO_3_S, 356.1320; found, 356.1322.

#### (Z)-*N*-Cyclohexyl-3-phenyl-2-((triphenylsilyl)oxy)acrylamide **2f**

White foam. *R*_*f*_ = 0.35 (PE/Et_2_O = 7:3). Yield: 65% (98 mg). ^1^H NMR: δ 7.57–7.54 [m, 6H], 7.46–7.40 [m, 2H], 7.36–7.28 [m, 9H], 7.07–6.97 [m, 3H], 6.84 [s, 1H], 6.16 [d br, *J* = 8.2 Hz, 1H], 3.66–3.52 [m, 1H], 1.65–1.47 [m, 5H], 1.22–0.85 [m, 3H], 0.64–0.47 [m, 2H]. ^13^C{^1^H} NMR: δ 163.7, 143.2, 135.6, 134.0, 132.7, 130.5, 129.7, 128.2, 128.0, 127.4, 116.8, 48.4, 32.5, 25.5, 25.0. HRMS (ESI) *m*/*z*: [M + H]^+^ calcd for C_33_H_34_NO_2_S, 504.2348; found, 504.2367.

#### (*Z*)-*N*-Cyclohexyl-4-phenyl-2-((triphenylsilyl)oxy)but-2-enamide **2g**

White solid, mp = 99.1–102.3 °C. *R*_*f*_ = 0.30 (PE/Et_2_O = 7:3). Yield: 65% (101 mg). ^1^H NMR: δ 7.67 [dd, *J* = 8.0, 1.5 Hz, 6H], 7.54–7.38 [m, 9H] 7.19–7.07 [m, 3H], 6.82 [dd, *J* = 7.6, 1.8 Hz, 2H], 6.17 [t, *J* = 7.6 Hz, 1H], 6.16 [d, *J* = 7.6 Hz, 1H], 3.62–3.59 [m, 1H], 3.15 [d, *J* = 7.5 Hz, 2H], 1.61–1.49 [m, 5H], 1.30–1.12 [m, 2H], 1.04–0.84 [m, 1H], 0.63–0.36 [m, 2H]. ^13^C{^1^H} NMR: δ 162.8, 143.2, 139.5, 135.6, 132.7, 130.9, 128.5 (×2), 128.4, 126.1, 117.9, 48.0, 32.6, 32.3, 25.6, 24.9.HRMS (ESI) *m*/*z*: [M + H]^+^ calcd for C_34_H_36_NO_2_Si, 518.2510; found, 518.2521.

#### (*Z*)-*N*-Cyclohexyl-4-phenyl-2-((triphenylsilyl)oxy)but-2-enamide **2h**

Colorless oil. *R_f_* = 0.36 (PE/EtOAc = 4:1). Yield: 71% (109 mg). ^1^H NMR: δ 7.67–7.60 [m, 6H], 7.52–7.37 [m, 9H], 6.17 [d, *J* = 8.4 Hz, 1H], 6.01 [t, *J* = 7.6 Hz, 1H], 3.69–3.50 [m, 1H], 1.79 [q, *J* = 7.0 Hz, 2H], 1.64–1.45 [m, 4H], 1.31–1.13 [m, 5H], 1.01 [s, 7H], 0.81 [t, *J* = 7.2 Hz, 3H], 0.64–0.49 [m, 2H]. ^13^C{^1^H} NMR: δ 163.1, 142.5, 135.6, 132.9, 130.7, 128.3, 120.0, 47.9, 32.6, 31.7, 29.0, 28.6, 26.4, 25.6, 24.9, 22.7, 14.2. HRMS (ESI) *m*/*z*: [M + H]^+^ calcd for C_33_H_42_NO_2_Si, 512.2979; found, 512.2983.

#### (*S*,*Z*)-*tert*-Butyl (5-(Benzylamino)-5-oxo-4-((triphenylsilyl)oxy)pent-3-en-2-yl)carbamate **2i**

Colorless oil. *R_f_* = 0.24 (PE/EtOAc = 4:1). [α]_D_ = +20.6 (*c* 1.0, CHCl_3_). Yield: 40% (66 mg). ^1^H NMR: δ 7.60 [d, *J* = 8.1 Hz, 6H], 7.49–7.41 [m, 3H], 7.39–7.32 [m, 6H], 7.28–7.15 [m, 3H], 6.96–6.87 [m, 2H], 6.52 [s, 1H], 5.86 (d, *J* = 9.7 Hz, 1H), 4.35–3.99 [m, 4H], 1.39 [s, 9H], 0.68 [d, *J* = 6.4 Hz, 3H]. ^13^C{^1^H} NMR: δ 163.8, 154.7, 142.5, 137.7, 135.6, 132.3, 130.9, 128.7, 128.4, 128.0, 127.5, 120.2, 79.2, 43.7, 43.3, 28.5, 20.7. HRMS (ESI) *m*/*z*: [M + H]^+^ calcd for C_33_H_39_N_2_O_4_Si, 555.2674; found, 555.2671.

#### (*Z*)-*N*-Cyclohexyl-3-(4-methoxyphenyl)-2-((triphenylsilyl)oxy)acrylamide **2l**

White foam. *R*_*f*_ = 0.41 (PE/EtOAc = 4:1). Yield: 65% (104 mg). ^1^H NMR: δ 7.64–7.26 [m, 17H], 6.80 [s, 1H], 6.52 [d, *J* = 8.8 Hz, 2H], 6.13 [d, *J* = 8.3 Hz, 1H], 3.72 [s, 3H], 3.67–3.49 [m, 1H], 1.66–1.44 [m, 5H], 1.31–1.09 [m, 2H], 1.01–0.84 [m, 1H], 0.64–0.48 [m, 2H]. ^13^C{^1^H} NMR: δ 163.9, 159.0, 141.9, 135.7, 132.9, 131.1, 130.5, 128.1, 126.6, 116.5, 113.5, 55.3, 48.3, 32.5, 25. 6, 25.0. HRMS (ESI) *m*/*z*: [M + H]^+^ calcd for C_34_H_36_NO_3_Si, 534.2459; found, 534.2467.

### Procedure for the Synthesis of Compound **3** in Flow

Product **2f** (0.06 mmol, 1 equiv, 0.01M), Umemoto II reagent (0.12 mmol, 2 equiv), and Ru(bipy)_3_Cl_2_ (1 mol %) were dissolved in acetonitrile (6 mL) under stirring. The solution was degassed with Ar for 5 min, then loaded into the 6 mL loading loop, and eluted at 1.5 mL h^–1^ (residence time 2 h) for a total of 12 h to ensure complete collection of the irradiated solution. The collected solution was evaporated under reduced pressure; then, the crude was dissolved in EtOAc and washed twice with water and once with brine. The combined organic layers were dried over anhydrous Na_2_SO_4_ and then eventually purified by flash chromatography (SiO_2_, PE → PE/EtOAc 10:1) to afford 6 mg of the desired product (32% yield).

#### *N*-Cyclohexyl-4,4,4-trifluoro-2-oxo-3-phenylbutanamide **3**

Colorless oil. ^1^H NMR: δ 7.46–7.34 [m, 5H], 6.76 [br s, 1H], 5.69 [q, *J* = 8.8 Hz, 1H], 3.74–3.51 [m, 1H], 1.92 [d, *J* = 12.1 Hz, 2H], 1.79–1.61 [m, 2H], 1.41–1.09 [m, 6H]. ^13^C{^1^H} NMR: δ 189.9, 157.3, 130.2, 129.4, 129.2, 127.6, 53.2 [q, *J* = 28.1 Hz], 48.9, 32.5, 25.2, 24.6 (CF_3_ is not visible). ^19^F NMR (decoupled) δ −66.5. HRMS (ESI) *m*/*z*: [M + H]^+^ calcd for C_16_H_18_F_3_NO_2_, 313.1290; found, 313.1279.

### Procedure for the Synthesis of Compound **4** in Flow

4-Methoxybenzenediazonium tetrafluoroborate (0.20 mmol, 1 equiv, 0.01 M), crude product **2g** (assumed 0.20 mmol, 1 equiv) and Ru(bipy)_3_Cl_2_(1 mol %) were dissolved in acetone (20 mL) under stirring. The solution was degassed with Ar for 5 min, then loaded into the 20 mL loading loop, and eluted at 3 mL h^–1^ (residence time 1 h) for a total of 10 h to ensure complete collection of the irradiated solution. Prior to irradiation, the flow apparatus was flushed with nitrogen and conditioned with degassed acetone (including the airtight syringe). The collected solution was evaporated under reduced pressure and purified by flash chromatography (SiO_2_, PE/EtOAc 4:1) to afford 47 mg of the final product (75% yield).

#### 3-Benzyl-*N*-cyclohexyl-2,5-dioxohexanamide **4**

Colorless oil. *R_f_* = 0.48 (PE/EtOAc = 3:1). ^1^H NMR: δ 7.32–7.27 [m, 2H], 7.24–7.19 [m, 3H], 6.78 [d, *J* = 9.8 Hz, 1H], 4.08 [tt, *J* = 9.5, 4.4 Hz, 1H], 3.77–3.70 [m, 1H], 3.12 [dd, *J* = 13.4, 4.8 Hz, 1H], 2.94 [dd, *J* = 18.5, 10.2 Hz, 1H], 2.63 [dd, *J* = 18.5, 4.1 Hz, 1H], 2.49 [dd, *J* = 13.5, 9.4 Hz, 1H], 2.06 [s, 3H], 2.00–1.87 [m, 2H], 1.78–1.60 [m, 3H], 1.42–1.31 [m, 4H], 1.22–1.18 [m, 1H]. ^13^C{^1^H} NMR: δ 206.6, 200.2, 158.8, 138.3, 129.1, 128.6, 126.7, 48.5, 44.8, 41.8, 36.6, 32.7, 32.6, 29.4, 25.4, 24.7. HRMS (ESI) *m*/*z*: [M + H]^+^ calcd for C_19_H_26_NO_3_, 316.1907; found, 316.1920.

## Supporting Information Available

Photochemical equipment, UV–vis characterization of diazoketones **1a**–**g**, determination of the quantum yield, and copies of NMR spectra (PDF)
