# Supplementary material for: Into the Blue: Ketene Multicomponent Reactions under Visible Light
Source: J Org Chem. 2021 Apr 6;86(8):5845–51. doi: 10.1021/acs.joc.1c00278 (PMC8154565; doi:10.1021/acs.joc.1c00278)

Equipment, procedures and experimental data

for the manuscript

Into the Blue: Ketene Multicomponent Reactions under Visible Light

Pietro Capurro, Chiara Lambruschini, Paola Lova, Lisa Moni and Andrea Basso*

List of contents

Photochemical equipment **S2**
Determination of the quantum yield for a model K-3CR **S4**
UV-Vis characterization of diazoketones 1a-g and reaction mixtures **S7**
Copies of NMR spectra **S12**

Photochemical equipment

A) Batch Photoreactor

Batch reactions were conducted in a dedicated apparatus consisting of an aluminum cooling plate (LWH 160x100x25 mm) with 6 OSRAM® Oslon SSL 80 LDCQ7P (nominal 450 nm, royal blue) LEDs mounted in series powered by a MeanWell® LPC-20-700 constant current power supply (700 mA) and a water-cooled aluminum vessel holder. The vessel holder (LWH 170x110x38 mm) holds the sealed vials 10 mm over the LEDs in a fixed position while cooling both the LED plate and the vial, keeping the temperature of the latter below 20°C. Both the LED plate and the water-cooled vessel were custom built, while the vials were purchased from Wicom International Co. (WIC43005, 5 mL crimp top vial 38.5x22.0 mm; WIC44510 20 mm crimp caps with 3.0 mm PTFE septum).

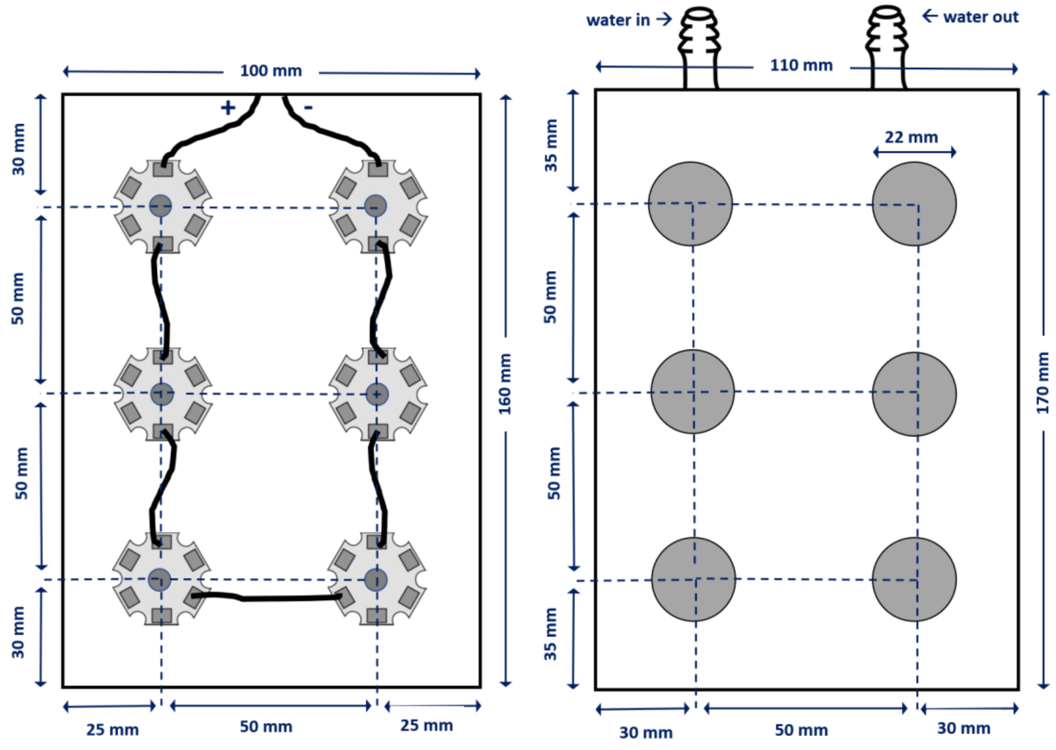

B) Flow Photoreactor

Flow reactions were conducted in a custom-built flow system consisting of:

- A programmable syringe pump (*World Precision Instrument AL-1000*);
- 1-50 mL air-tight syringes (purchased from *SGE Analytical / Trajan Scientific and Medical*);
- Loading loops (6 or 20 mL) in FEP tubing (ID 1.6 mm, OD 3.2 mm);
- Reaction coil (3 mL) in FEP tubing (ID 0.8 mm, OD 1.6 mm);
- Irradiation system with 12 x OSRAM® Oslon SSL 80 LDCQ7P (nominal 450 nm, royal blue) LEDs mounted in series powered by a MeanWell® LPC-35-700 constant current power supply (700 mA)
- A light-shielded collecting flask

All tubing was purchased from BOLA (Bohlender GmbH) and after mounting was shielded with aluminum foil to prevent undesired light exposure (except for the reaction coil). The reaction coil (height 70 mm) consists of FEP tubing for a total volume of 3 mL wrapped around a 50 mm cylindrical glass water jacket to

ensure proper cooling, keeping the reaction mixture below 20°C during the irradiation. The reaction coil is clamped to a support and is placed in the middle of the irradiation system, with the tubing spacing 35 mm from the nearest LEDs plate. The irradiation system consists of 4 aluminum LEDs plates connected in series and mounted on a 200x200 mm wooden support. Each aluminum cooling plate (HWD 130x40x25 mm) bears 3 blue LEDs (40 mm apart) and is placed in the middle of each side of the square wooden support. To ensure proper heat dispersion of the LEDs plates, an 80 mm fan (powered independently with a 12V DC power supply) is placed in the middle of the wooden support in a hollow cavity to grant air circulation.

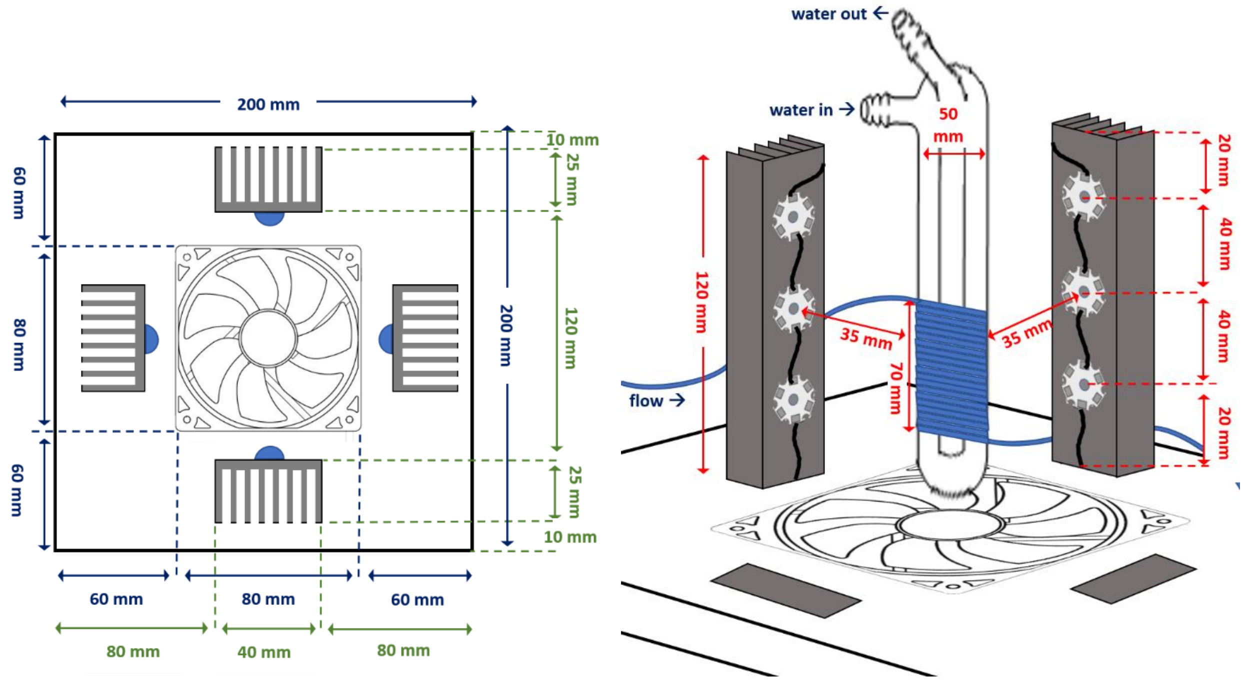

Determination of the quantum yields for a model K-3CR

The quantum yield for the Wolff rearrangement and the multicomponent process of the model reaction depicted in Scheme S1 has been determined. The reaction quantum yield (ϕ) was calculated as

$$\Phi = \frac{N.\ of\ consumed\ or\ produced\ molecules}{N.\ of\ absorbed\ photons}$$

The number of consumed and produced molecules was retrieved from the NMR monitoring of the model reaction depicted in Scheme S1, while the number of absorbed photons was estimated employing a fiber-based optical setup built *ad hoc*. The photon flow from the LED system was indeed collected using an integrating sphere (Avasphere, 5 cm, Avantes) opportunely placed around the reaction vial and connected to a CMOS spectrometer (AvaSpec-ULS2048CL-EVO, Avantes, resolution 1.4 nm) with an optical fiber. The estimation of the emitted number of photons was allowed by a precise calibration of the set-up response using a calibrated white light source (AvaLight-HAL-CAL, Aventes). The measurements were performed as follow.

a) *<u>Calibration procedure</u>*

To calibrate the set-up, the intensity emitted from the white light calibrated source was measured in the geometry employed for the reaction to account for possible losses. The measurement serves to relate the intensity collected by the detector, which is affected by the response of the detector itself, by the losses occurring in the sphere and in the optical fiber and by the reactor geometry, to the lamp power (Js^{-1}) previously calibrated by the manufacturer. The calibration was further confirmed trough comparison of the power of the white light and different LEDs measured using a power meter (PM100D mounting a S121C sensor, Thorlabs).

b) *<u>Measurement of absorbed photons</u>*

The calibration allowed to correlate the LED intensity to the emitted power. The emitted energy (J) was then estimated by integration of the emitted power over the time. The number of photon (N_P) was then calculated as the ratio between the energy emitted by the LED ($E_{emitted}$) and the energy of a photon ($E_{photon} = \frac{hc}{\lambda}$, where *h* is the Plank constant and *c* is the speed of light).

To estimate the number of photons absorbed by diazoketone **1a** accounting for all the losses of the reaction geometry including reflectance from the vial walls and refraction phenomena, we performed two measurements. The first consisted in the collection of LED light transmitted through the reaction vial containing all the reagents but the absorbing species **1a** in the very same experimental condition employed for the reaction. The second was the intensity transmitted through the vials during the reaction itself. The absorbed intensity was then calculated as the difference between the two values and converted to photon number as previously discussed.

c) *<u>NMR monitoring of model K-3CR</u>*

The model reaction has been carried out in $CDCl_3$ in the very same conditions as a standard reaction (see procedure for K-3CR in batch) with the addition of 0.05 mmol of DMSO as internal standard for integration reference. Aliquots (100 μL) of the solution were sampled, diluted in an NMR tube to a total volume of 700 mL of $CDCl_3$ and analyzed via ^{1}H-NMR before the irradiation and at defined time intervals. The reaction was preformed and monitored twice.

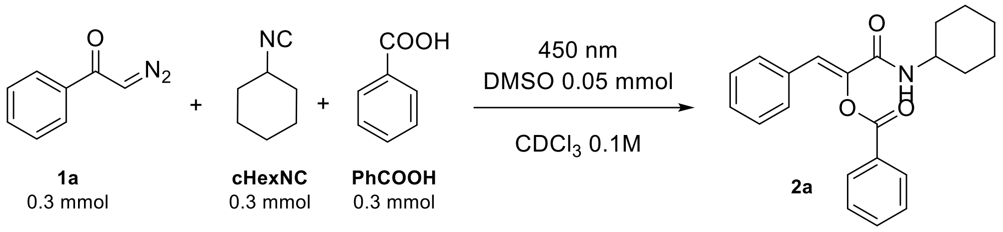

Scheme S1. Model Ketene Three Component Reaction (K-3CR) monitored for the determination of the quantum yield of the processes involved.

The first run was sampled every 15 minutes up to the complete disappearance of diazoketone **1a** (120 min); the second run was sampled every 5 minutes for 30 minutes. Integration of selected, non-interfered signals with respect to that of the internal standard (DMSO, 2.61 ppm, 1.000 H) allowed to calculate the rate of disappearance of the starting reagents (**1a**, **cHexNC** and **PhCOOH**) and the rate of formation of product **2a**. The results are reported in Table S1. Disappearance of diazoketone **1a**, cyclohexylisocyanide, benzoic acid and formation of product **2a** with respect to the irradiation time for both replicas are depicted in Figure S1.

Table S1. Disappearance rates of components and formation rate of the product for model reaction depicted in Scheme S1. Reference integration peak: DMSO (2.61 ppm, 1.000 H).
[a] monitored signal(s): δ 7.80 – 7.74 [m, 2H]
[b] monitored signal(s): δ 8.14 – 8.08 [m, 2H]
[c] monitored signal(s): δ 3.65 – 3.53 [m, 1H]
[d] monitored signal(s): δ 8.23 – 8.17 [m, 2H], 3.96 – 3.81 [m, 1H]

First replica				
**Time [min]**	**1a [%][a]**	**PhCOOH [%][b]**	**cHexNC [%][c]**	**2a [%][d]**
0	100.0	100.0	100.0	0.0
15	66.0	75.0	74.1	24.9
30	44.3	56.6	53.7	47.1
45	27.4	43.9	38.4	60.9
60	16.8	35.7	31.7	72.7
75	9.1	30.7	22.0	76.2
90	5.1	27.9	16.2	78.1
105	3.8	26.2	15.0	81.9
120	0.0	25.5	14.3	82.4
**Second replica**				
**Time [min]**	**1a [%][a]**	**PhCOOH [%][b]**	**cHexNC [%][c]**	**2a [%][d]**
0	100.0	100.0	100.0	0.0
5	84.8	88.3	89.8	9.1
10	74.3	79.4	79.7	18.2
15	64.8	72.7	74.0	26.5
20	55.8	65.8	67.1	34.0
25	46.7	58.5	60.5	40.0
30	39.7	52.9	54.6	45.6

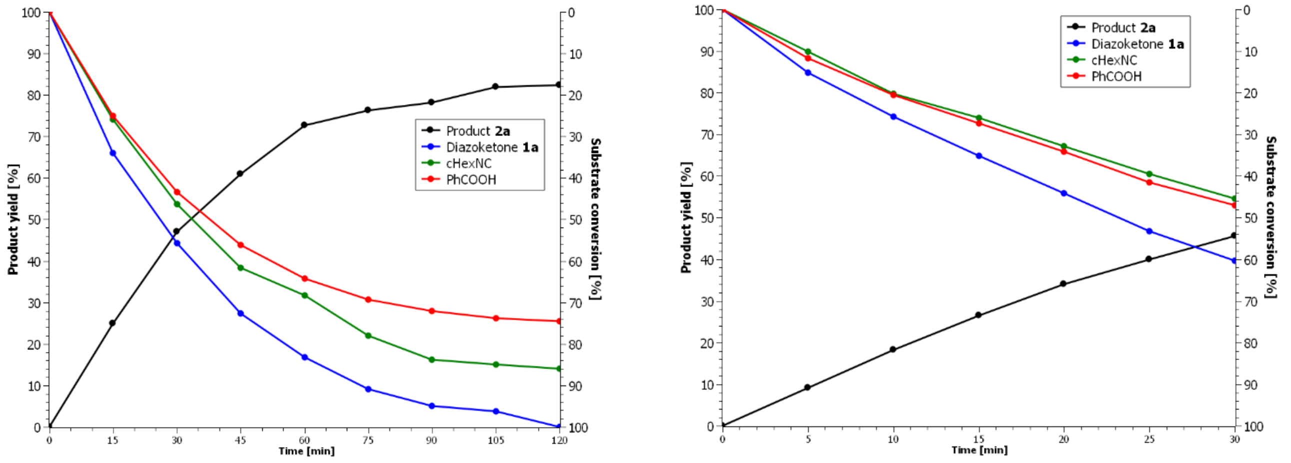

Figure S1. Disappearance of K-3CR components and formation of the product as function of the irradiation time. Left: first replica (sampling every 15 minutes for 120 minutes), right: second replica (sampling every 5 minutes for 30 minutes).

d) Determination of Wolff rearrangement quantum yield ϕ^{-1} and K-3CR quantum yield ϕ^{K3CR}

To minimize potential errors in the determination of the quantum yields of the processes due to the possible presence of undesired reactions, quantum yields were determined from the estimations of the absorbed photons and of the reacted species within the first 30 minutes of irradiation. Quantum yields for processes involved in the model reaction previously described are given by the following equations:

$$\Phi^{-1} = \frac{N.\ of\ consumed\ molecules\ of\ \mathbf{1a}}{N.\ of\ absorbed\ photons} = \frac{(1 - [\mathbf{1a}]) * 0.3\ mmol * N_A}{N_{photons}}$$

$$\Phi^{K3CR} = \frac{N.\ of\ molecules\ of\ \mathbf{2a}\ formed}{N.\ of\ absorbed\ photons} = \frac{[\mathbf{2a}] * 0.3\ mmol * N_A}{N_{photons}}$$

where [1a] and [2a] are the integral ratios of molecules **1a** and **2a** calculated at the NMR and N_A is Avogadro constant. Results are reported in Table S2.

Table S2. Disappearance rates of diazoketone **1a** and formation rate of the product **2a** for model reaction depicted in Scheme S1, number of photons absorbed by the reacting species **1a** and quantum yields ϕ^{-1}, ϕ^{K3CR} for the Wolff rearrangement and the K-3CR multicomponent process, respectively.
[a] data from NMR monitoring, second replica (section **c**, Table S1)
[b] calculated number of photons absorbed by the reacting species **1a** (calculated as described in section **b**)
[c] quantum yield for the Wolff rearrangement (value calculated at each time interval and mean ± standard deviation)
[d] quantum yield for the model Ketene 3-Component Reaction (value calculated at each time interval and mean ± standard deviation)

Time [min]	1a [%][a]	2a [%][a]	$N_{photons}$ [b]	ϕ^{-1} [c]	ϕ^{K3CR} [d]
0	100.0	0.0	1.24×10^{18}	---	---
5	84.8	9.1	2.60×10^{19}	1.05	0.63
10	74.3	18.2	5.00×10^{19}	0.93	0.66
15	64.8	26.5	7.29×10^{19}	0.87	0.66
20	55.8	34.0	9.47×10^{19}	0.84	0.65
25	46.7	40.0	1.15×10^{20}	0.83	0.63
30	39.7	45.6	1.35×10^{20}	0.80	0.61
				**0.89 ± 0.09**	**0.64 ± 0.02**

UV-Vis characterization of Diazoketones 1a-g

Compound 1a [0.1 mM in DCM]

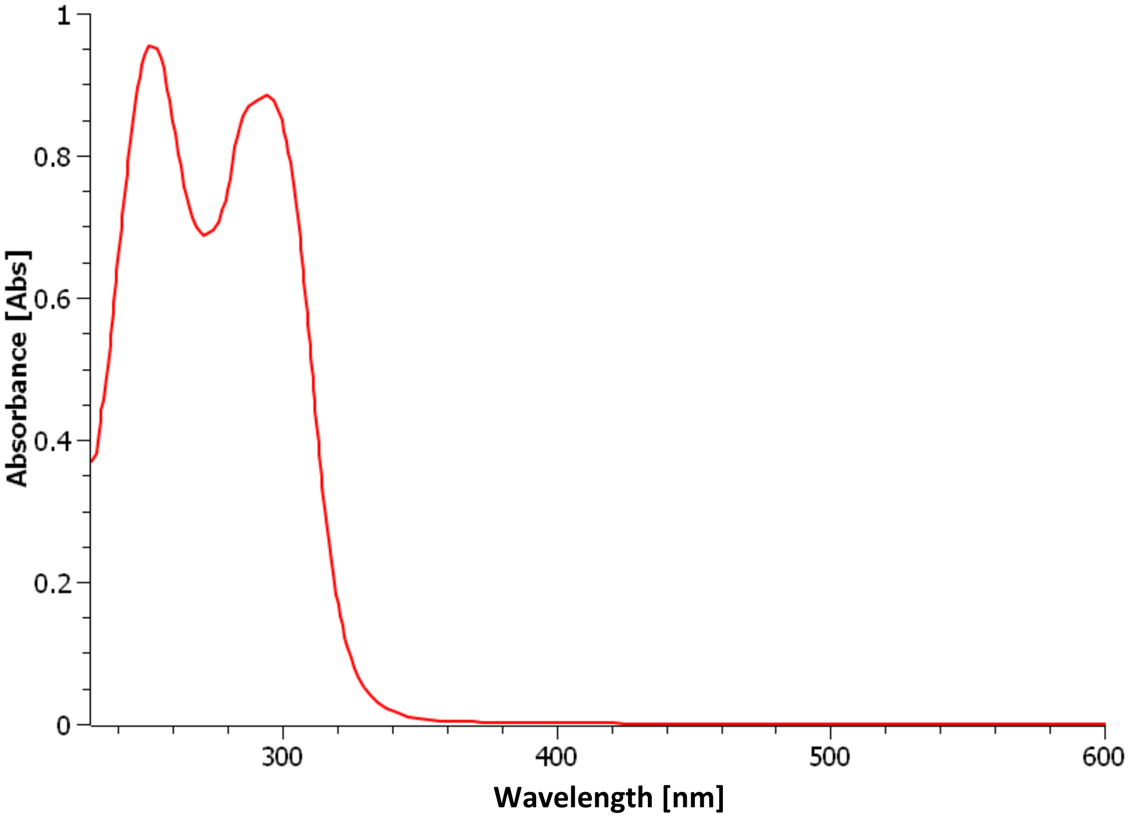

Compound 1b [0.1 mM in DCM]

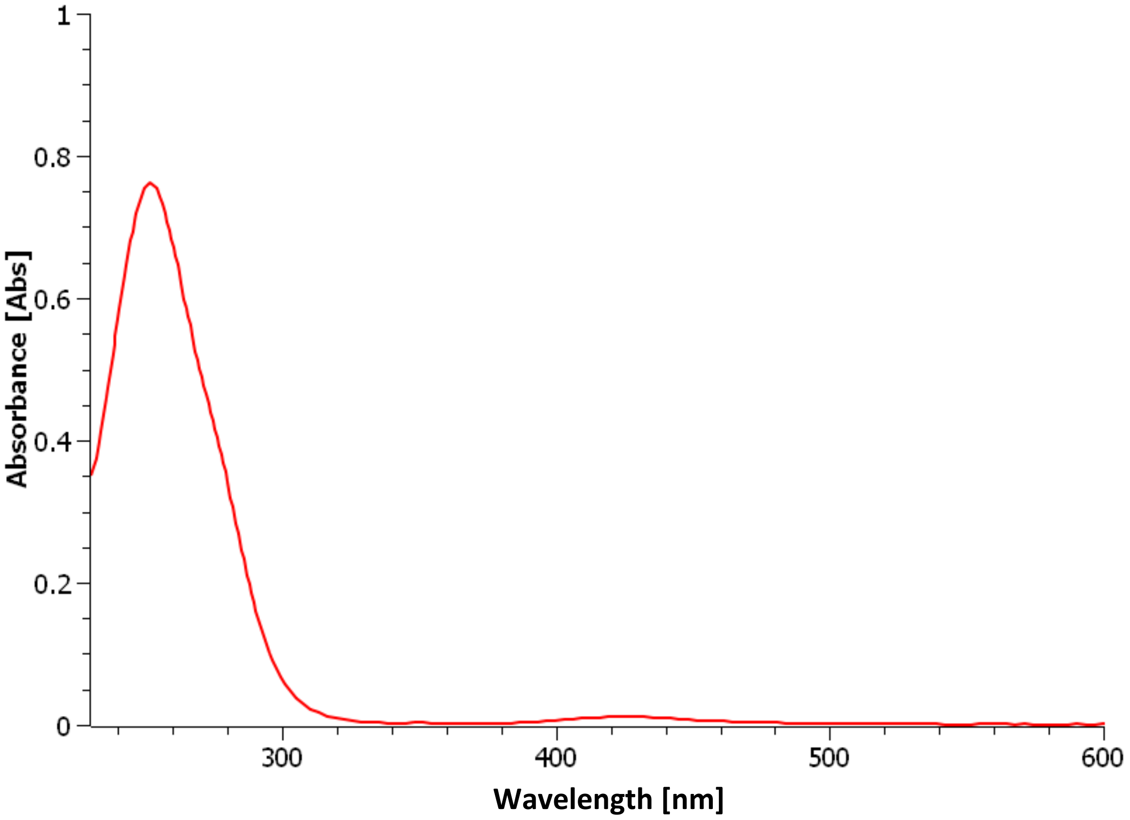

Compound 1c [0.05 mM in DCM]

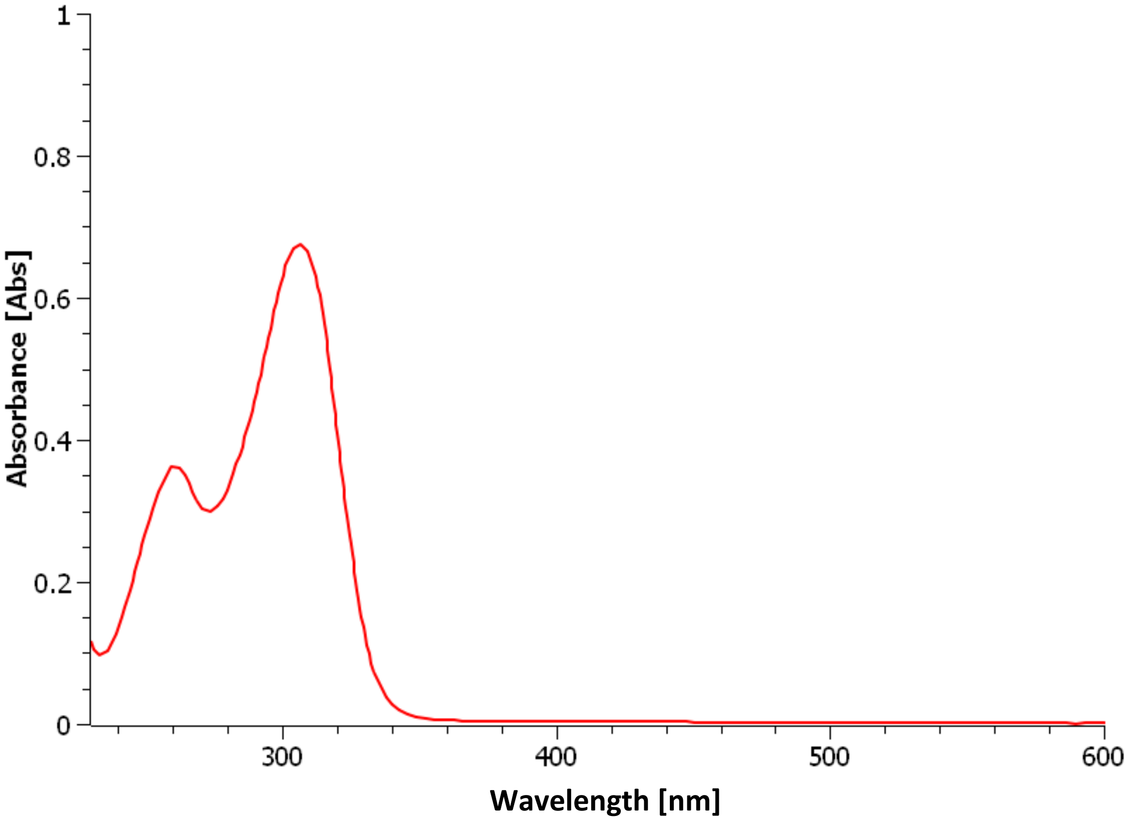

Compound 1d [0.1 mM in DCM]

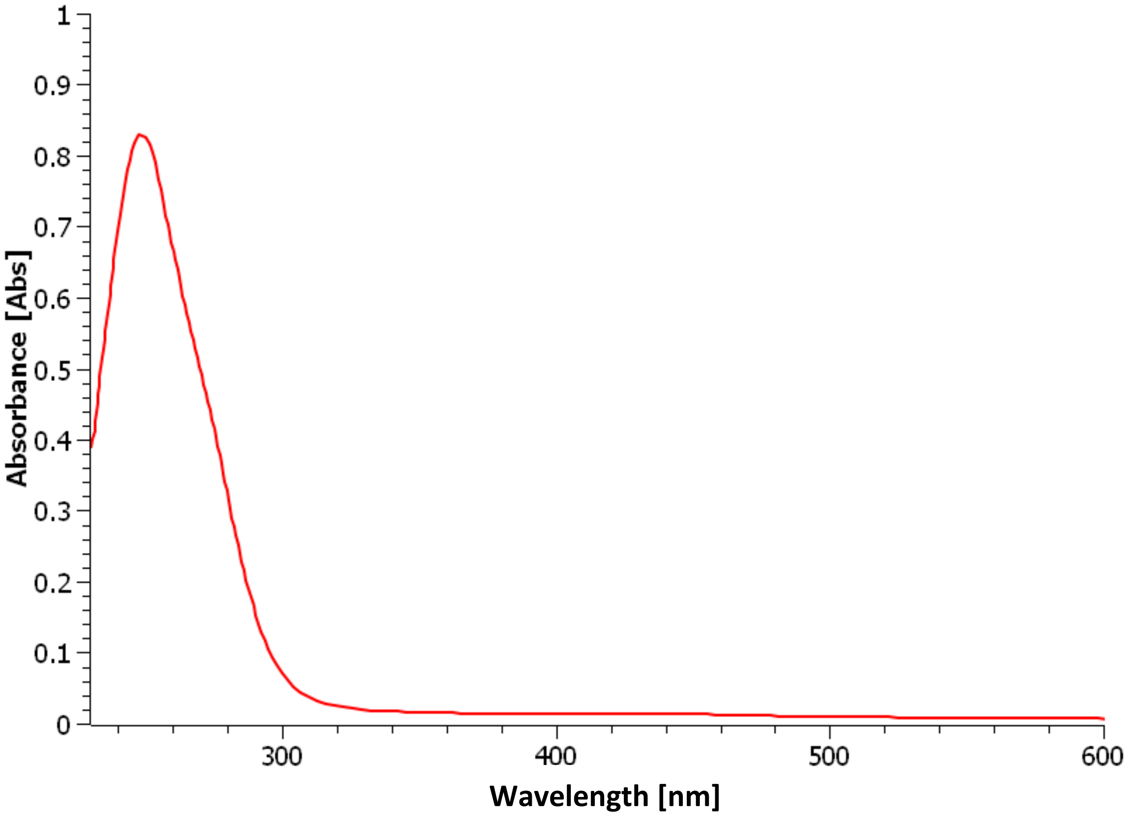

Compound 1e [0.1 mM in DCM]

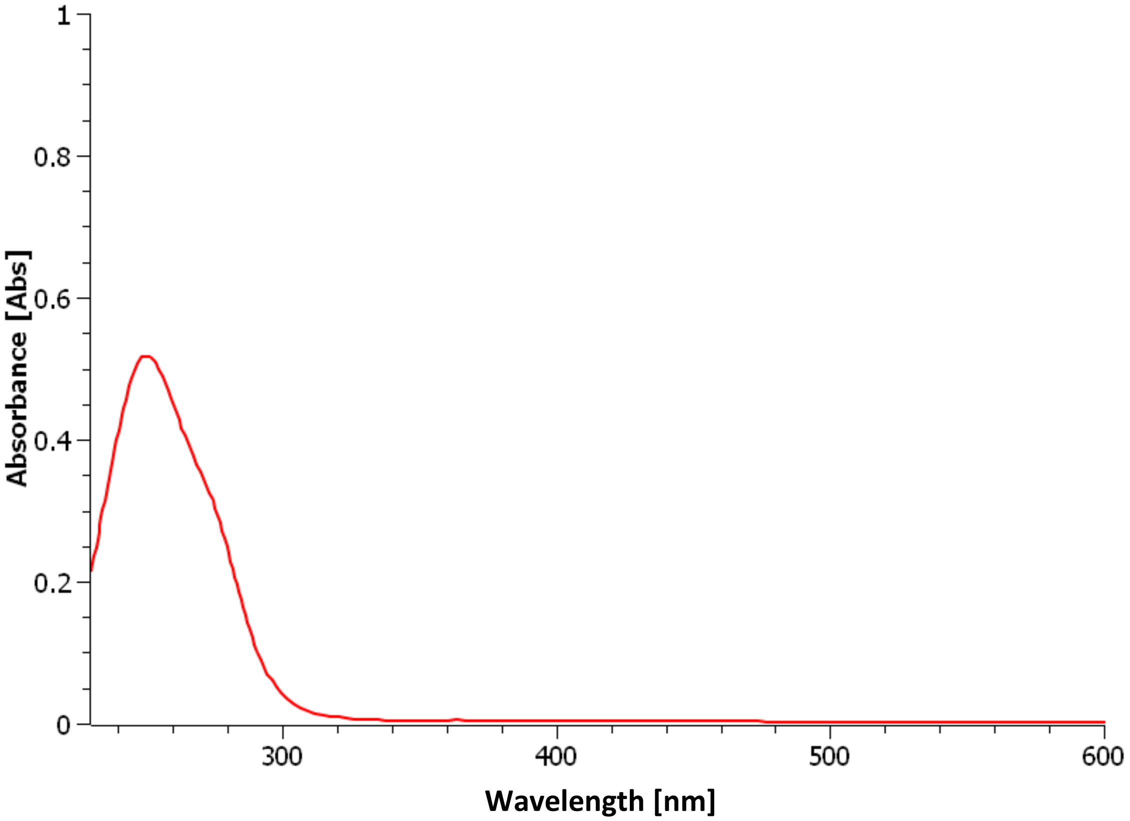

Compound 1f [0.05 mM in DCM]

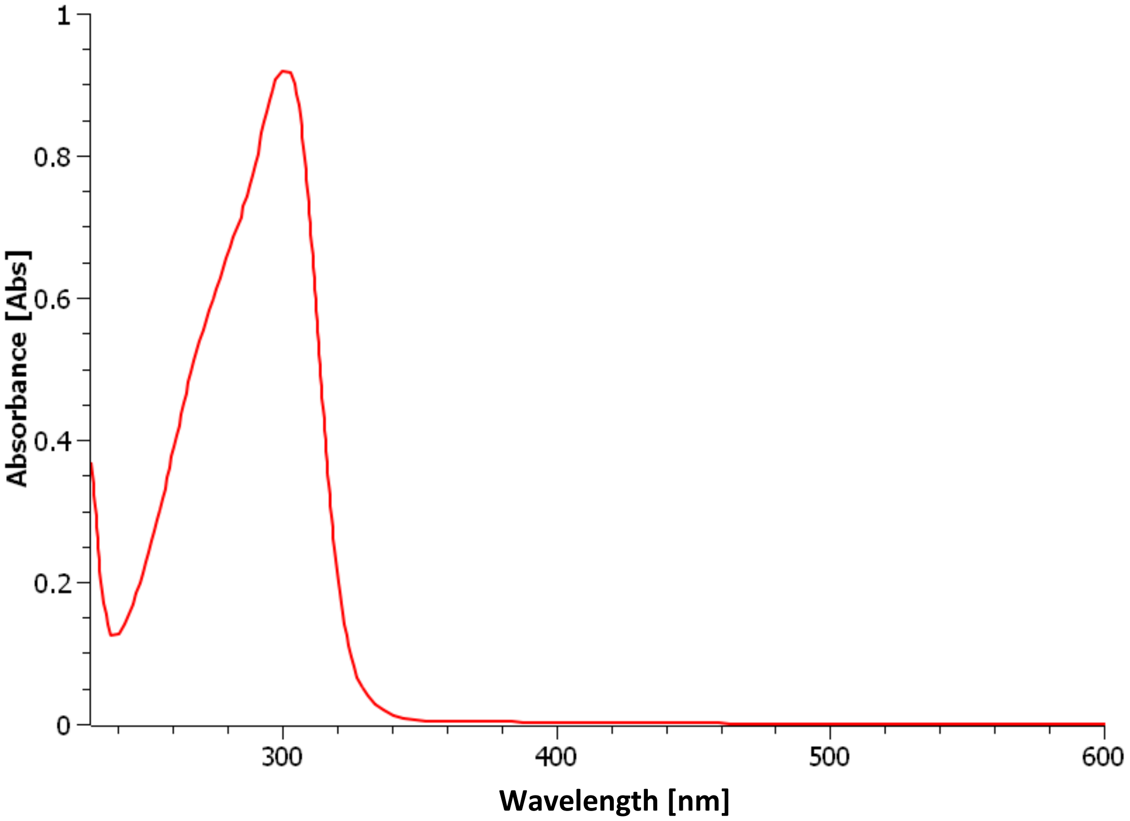

Compound 1g [0.05 mM in DCM]

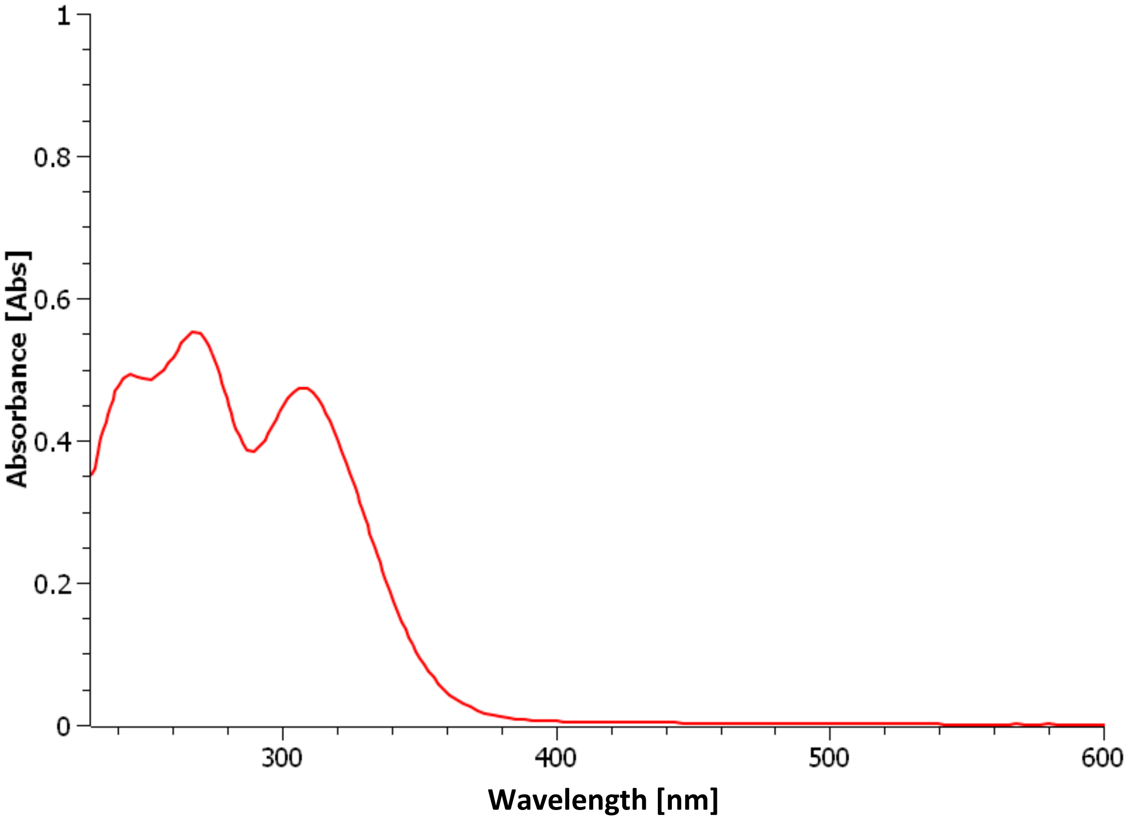

Detail of the absorption spectra of compounds 1a-g between 380-550 nm [0.1M in DCM] and their overlap with LEDs emission (normalised)

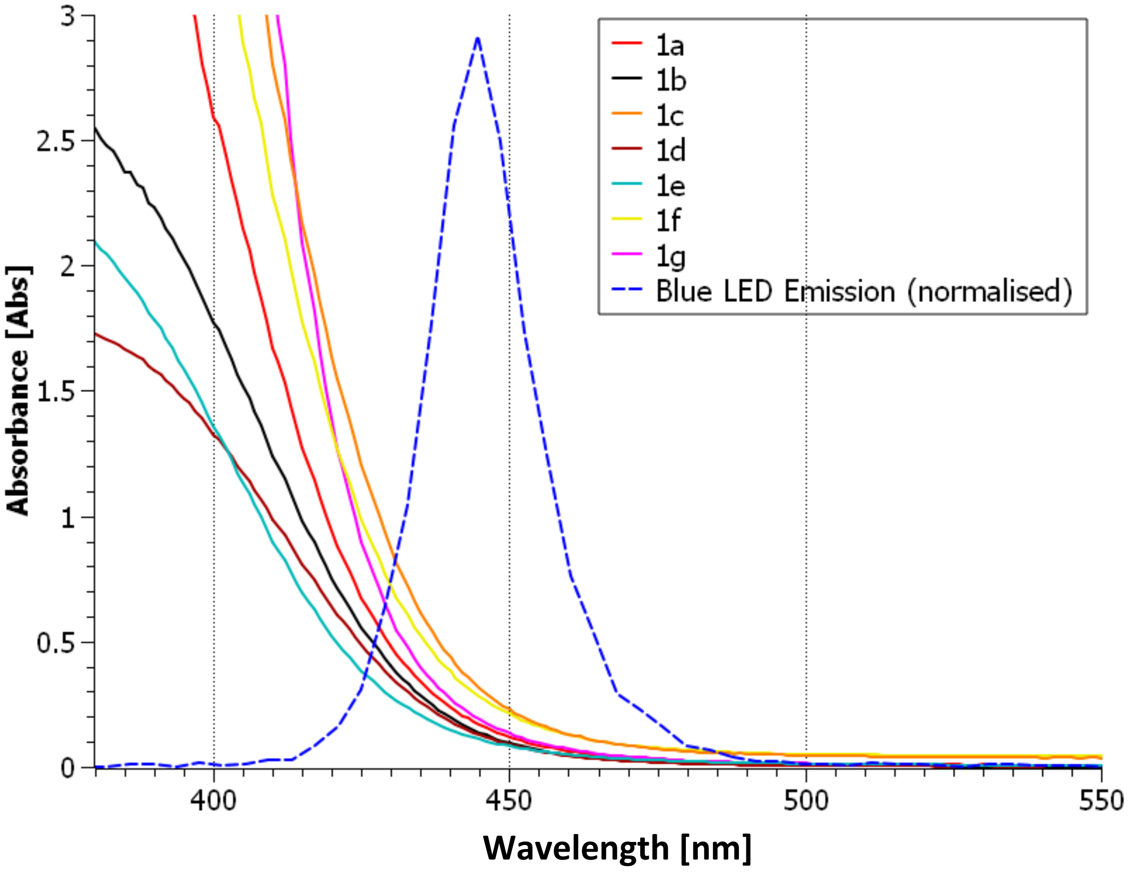

Spectra of single components and their mixtures in DCM 0.1M in the visible region [380 – 550 nm] and their overlap with LEDs emission (normalised)

K-3CR with **1b**, cHexNC and AcOH

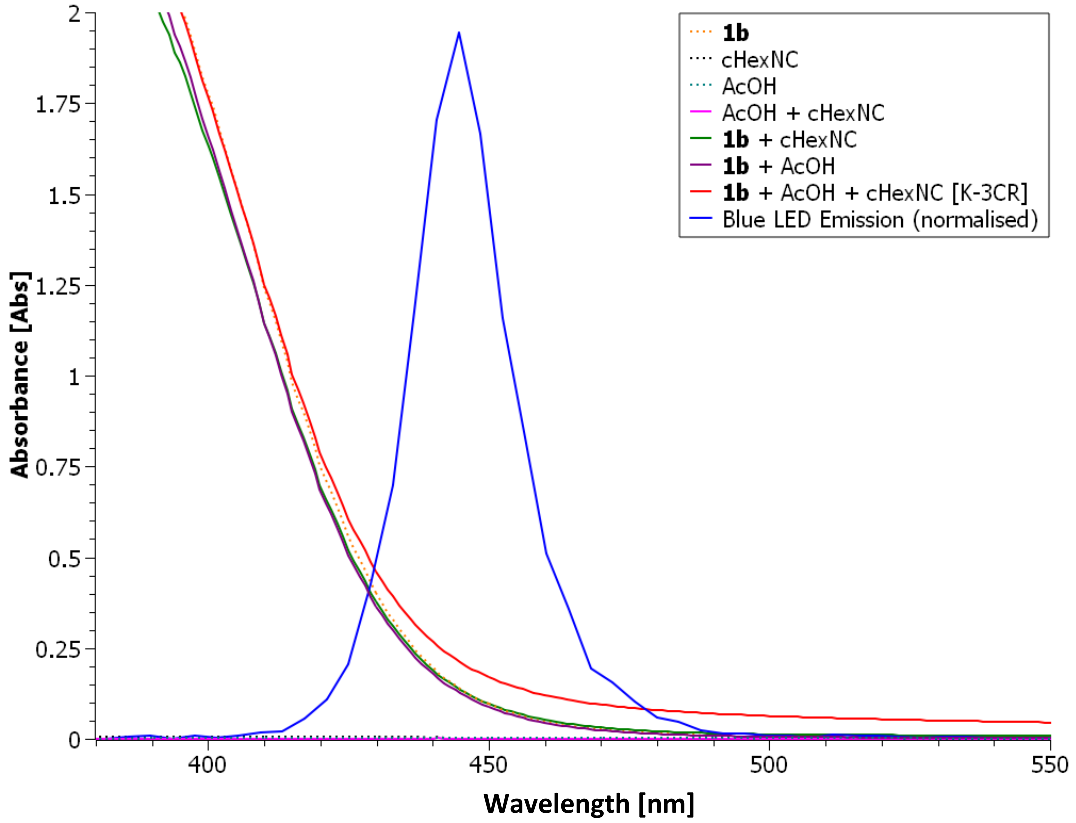

SK-3CR with **1b**, cHexNC and Ph_3SiOH

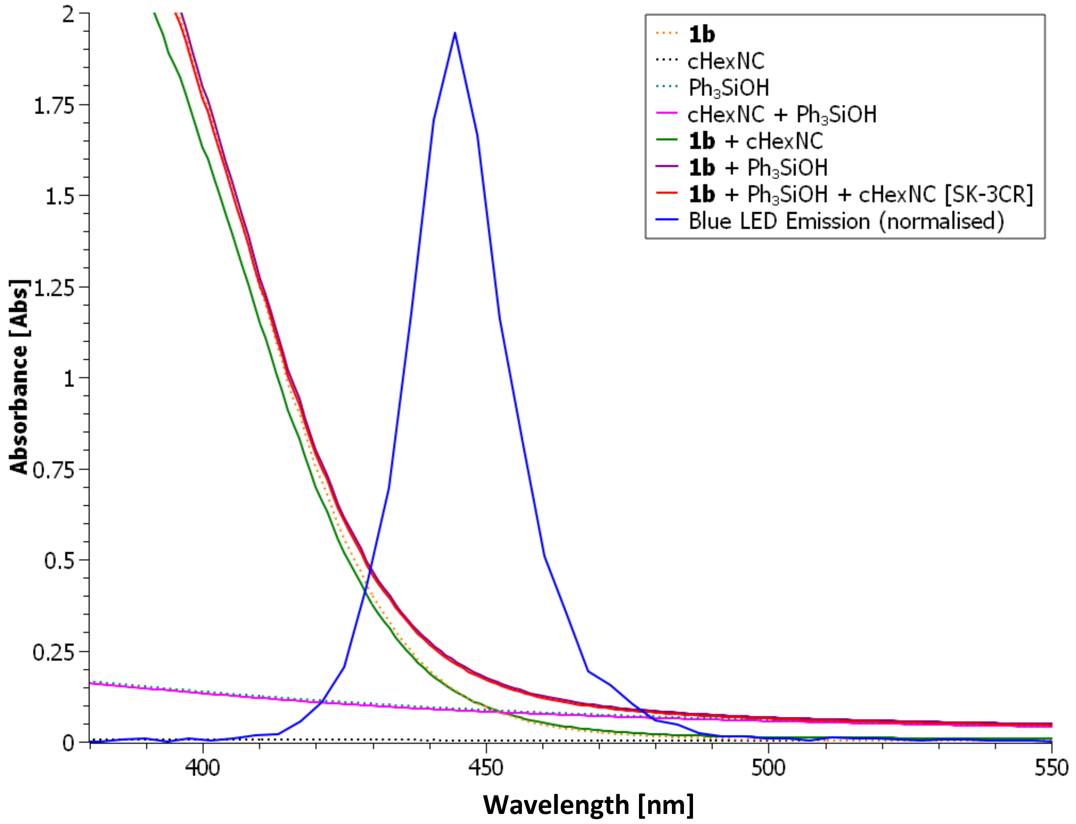

Copies of NMR spectra

Compound **2a**

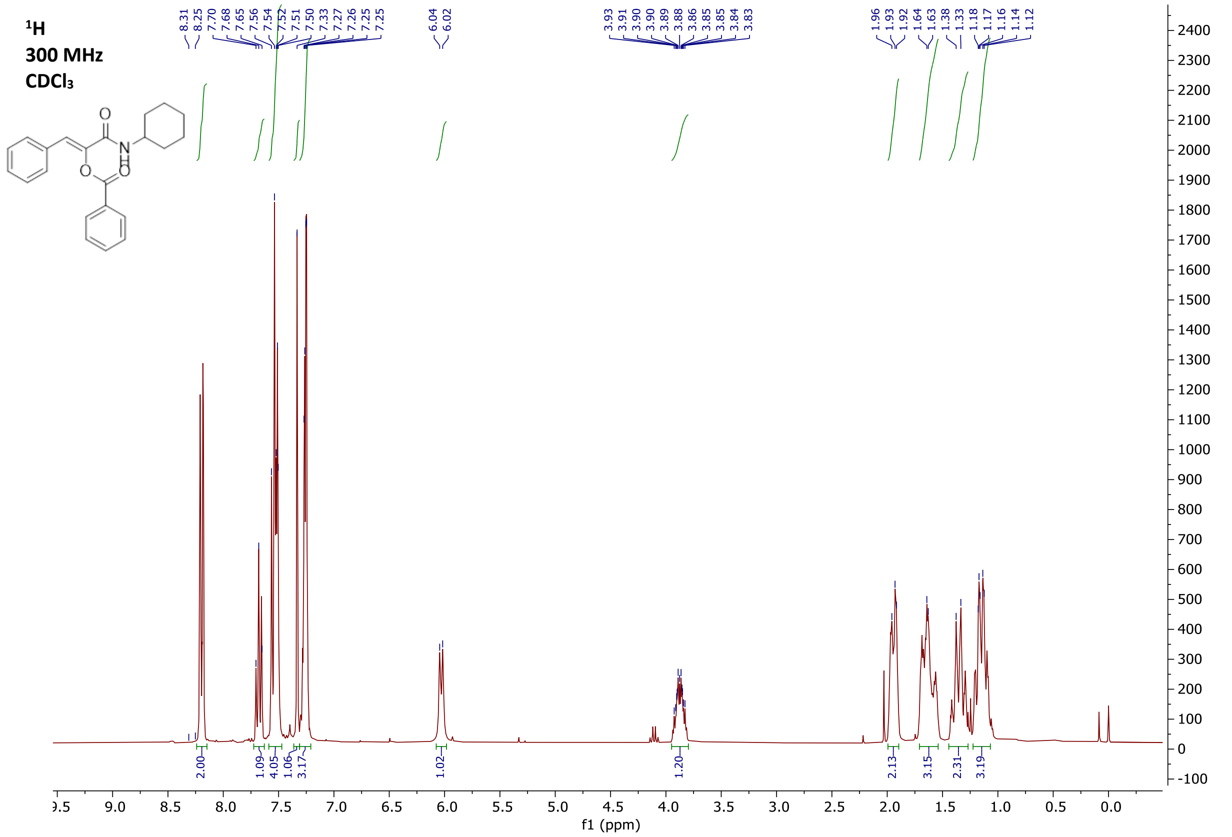

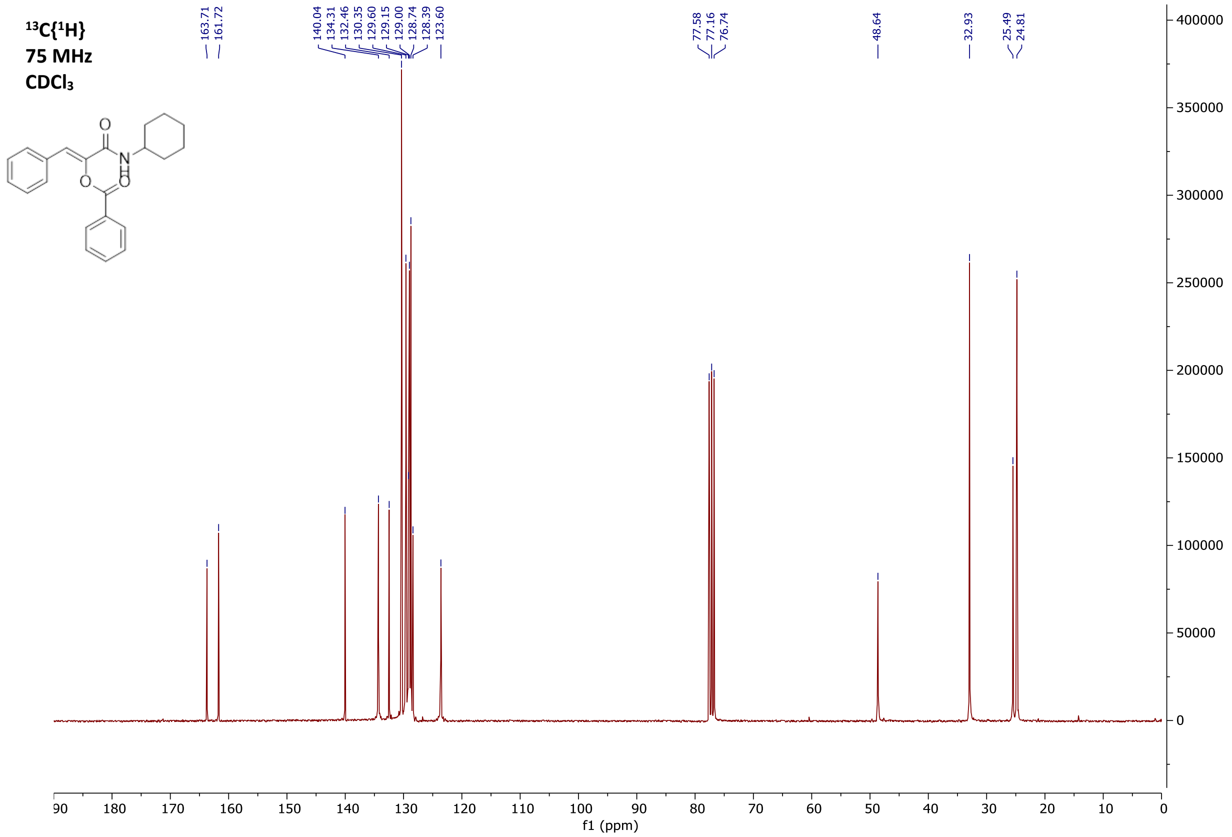

Compound **2b**

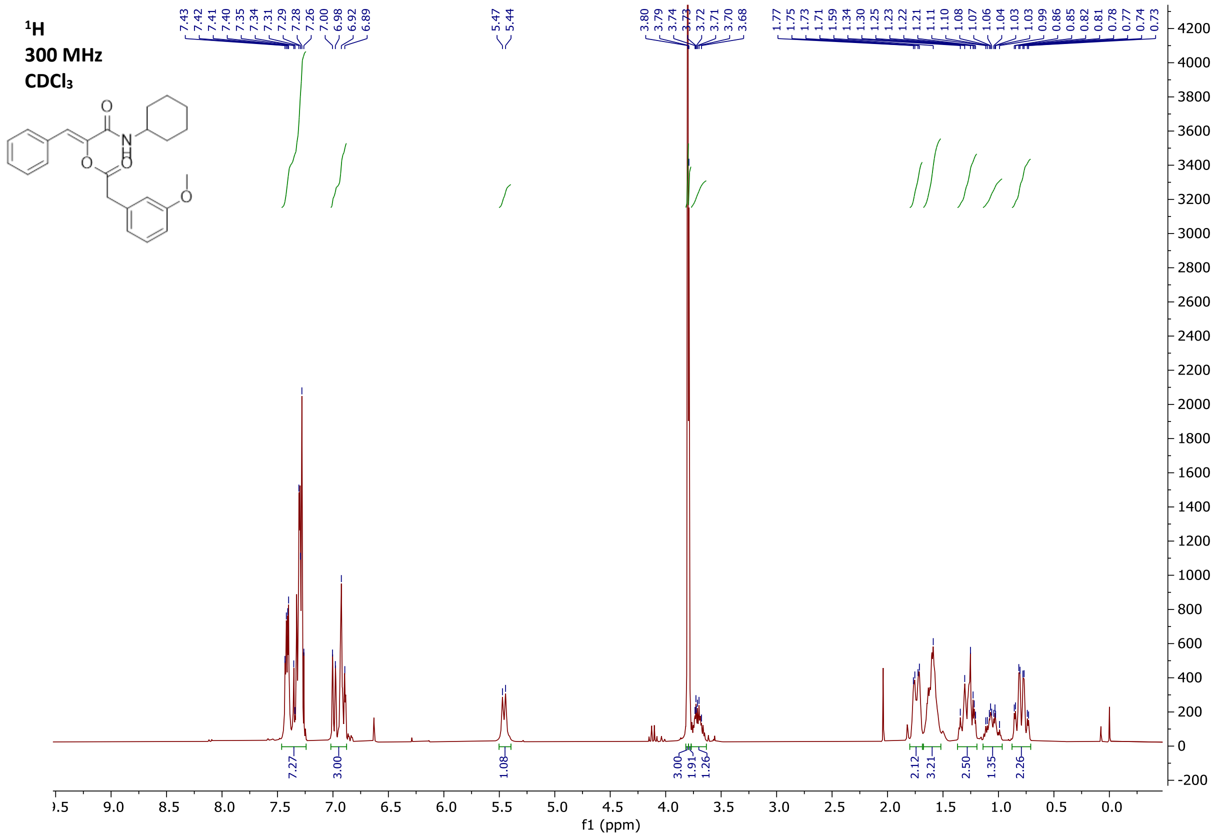

1H
300 MHz
CDCl3
O
N
H
O
O
O
7.43 7.42 7.41 7.40 7.35 7.34 7.31 7.29 7.28 7.26 7.00 6.98 6.92 6.89
5.47 5.44
3.80 3.79 3.74 3.73 3.72 3.71 3.70 3.68
1.77 1.75 1.73 1.71 1.59 1.34 1.30 1.25 1.23 1.22 1.21 1.11 1.10 1.08 1.07 1.06 1.04 1.03 1.03 0.99 0.86 0.85 0.82 0.81 0.78 0.77 0.74 0.73
7.27 3.00 1.08 3.00 1.91 1.26 2.12 3.21 2.50 1.35 2.26
f1 (ppm)

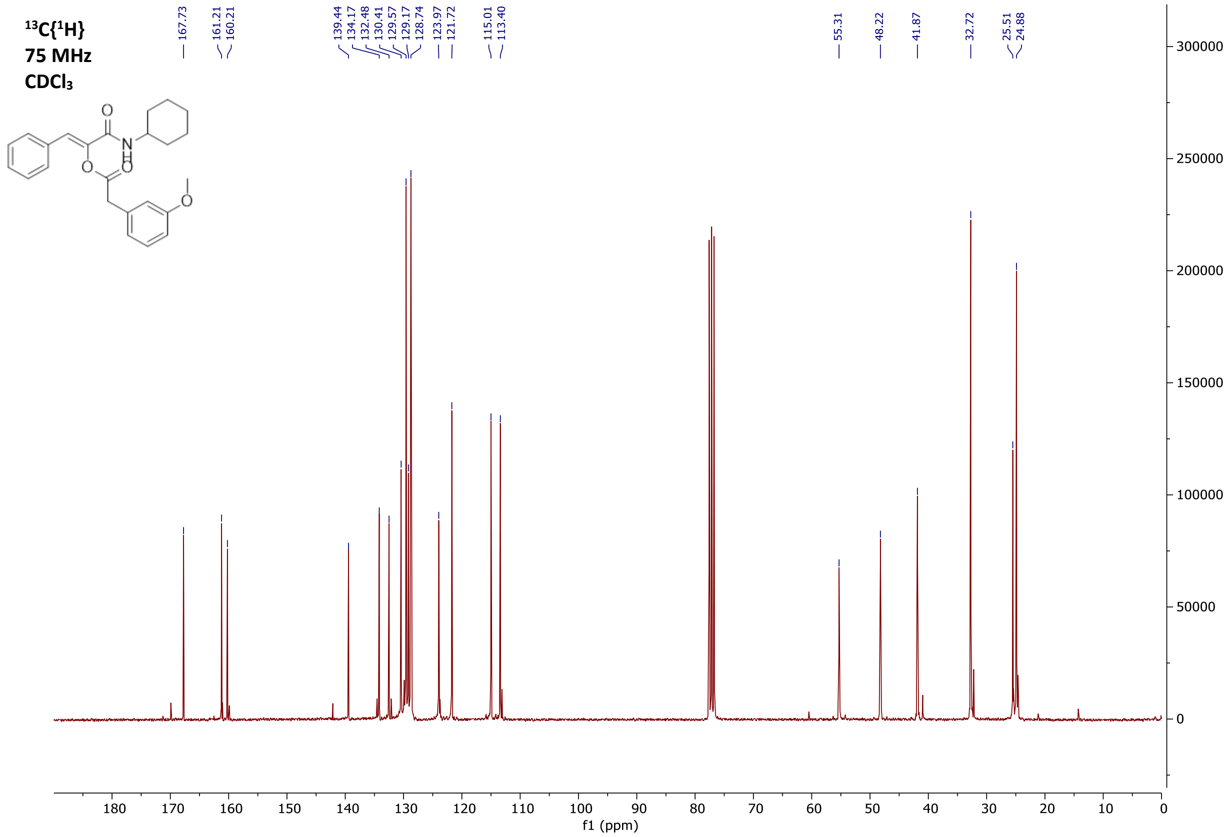

13C{1H}
75 MHz
CDCl3
167.73 161.21 160.21 139.44 134.17 132.48 130.41 129.57 129.17 128.74 123.97 121.72 115.01 113.40 55.31 48.22 41.87 32.72 25.51 24.88
f1 (ppm)

Compound **2c**

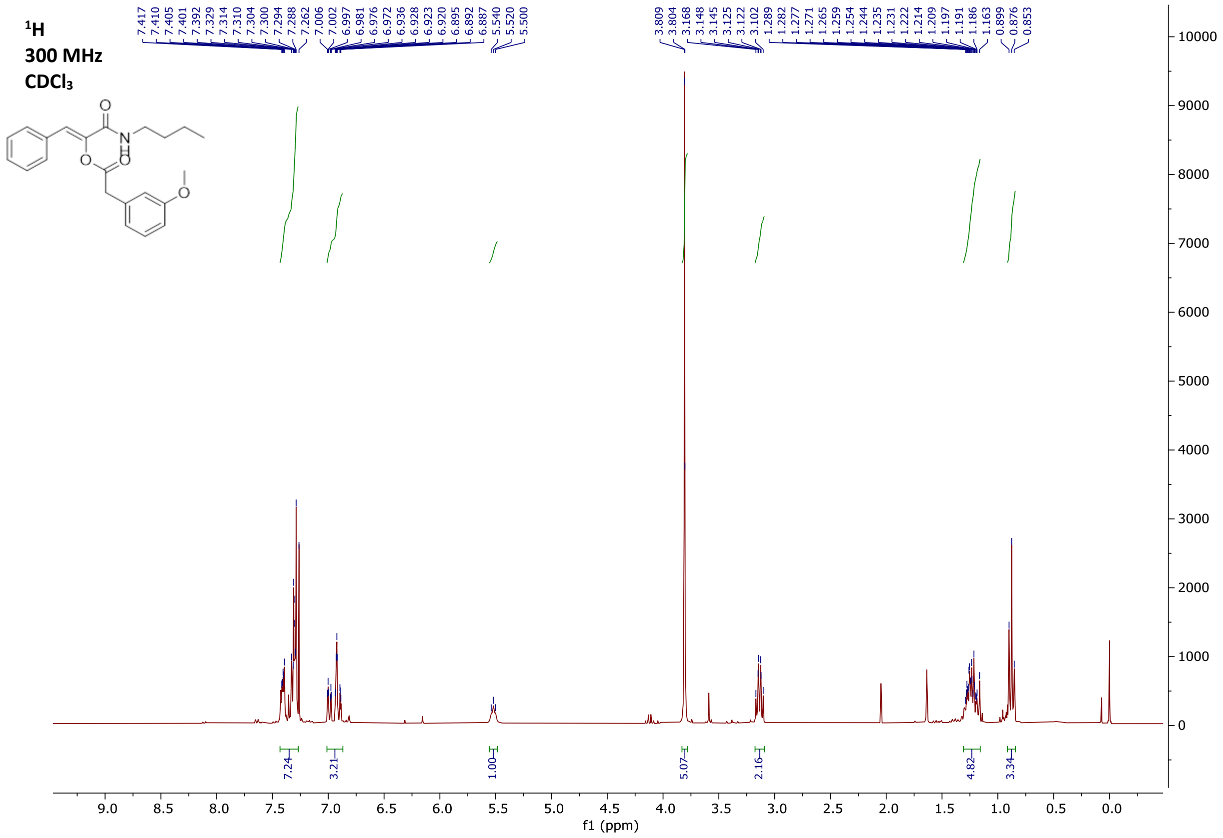

^{1}H
300 MHz
$CDCl_3$
7.417
7.410
7.405
7.401
7.392
7.329
7.314
7.310
7.304
7.300
7.294
7.288
7.262
7.006
7.002
6.997
6.981
6.976
6.972
6.936
6.928
6.923
6.920
6.895
6.892
6.887
5.540
5.520
5.500
3.809
3.804
3.168
3.148
3.145
3.125
3.122
3.102
1.289
1.282
1.277
1.271
1.265
1.259
1.254
1.244
1.235
1.231
1.222
1.214
1.209
1.197
1.191
1.186
1.163
0.899
0.876
0.853
7.24
3.21
1.00
5.07
2.16
4.82
3.34
f1 (ppm)

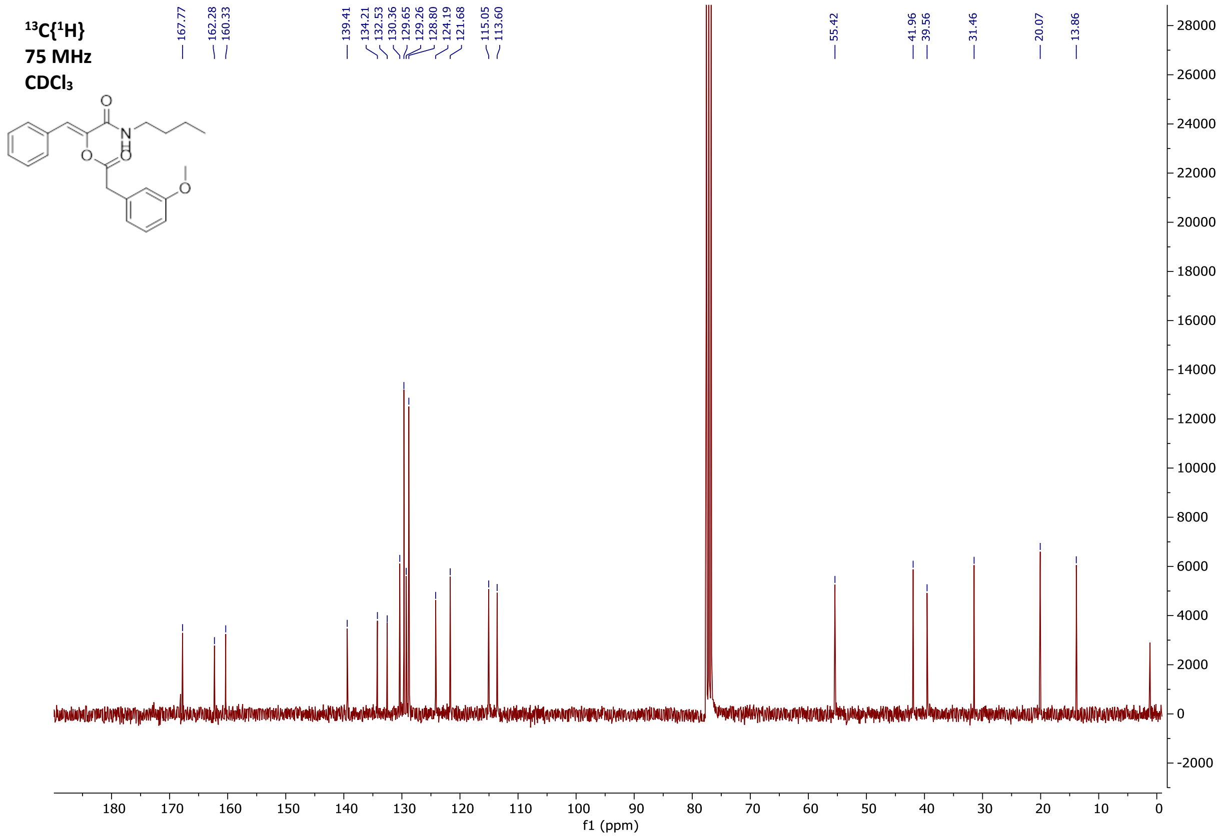

^{13}C{^{1}H}
75 MHz
$CDCl_3$
167.77
162.28
160.33
139.41
134.21
132.53
130.36
129.65
129.26
128.80
124.19
121.68
115.05
113.60
55.42
41.96
39.56
31.46
20.07
13.86
f1 (ppm)

Compound *(E)*-2d

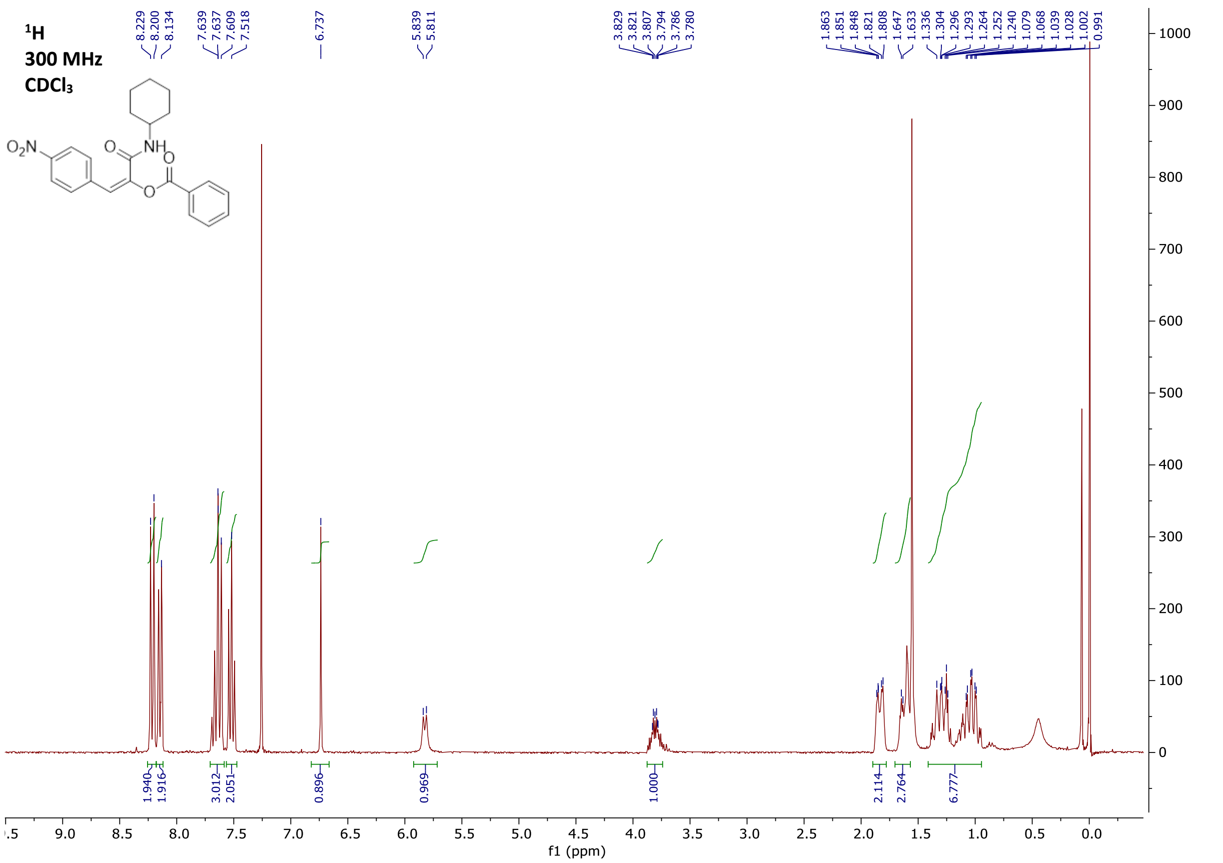

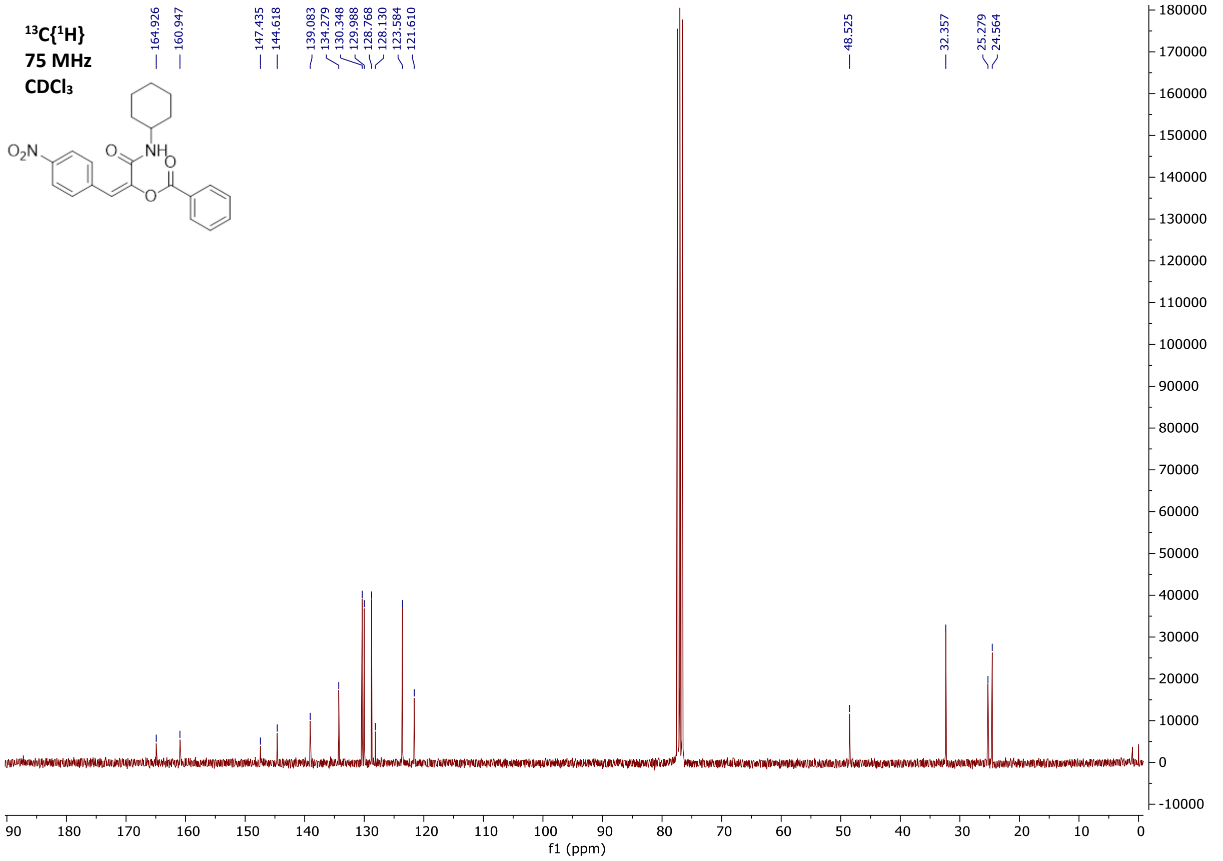

Compound ***(Z)*-2d**

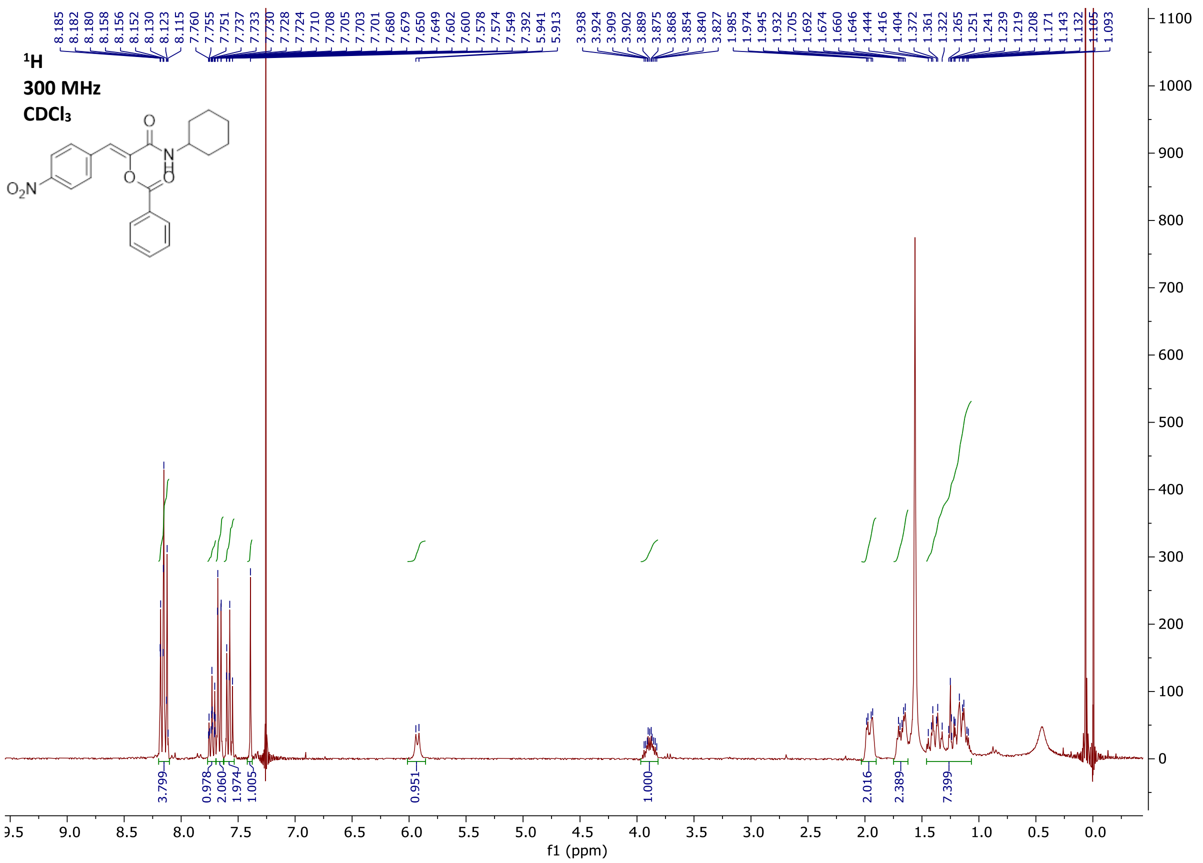

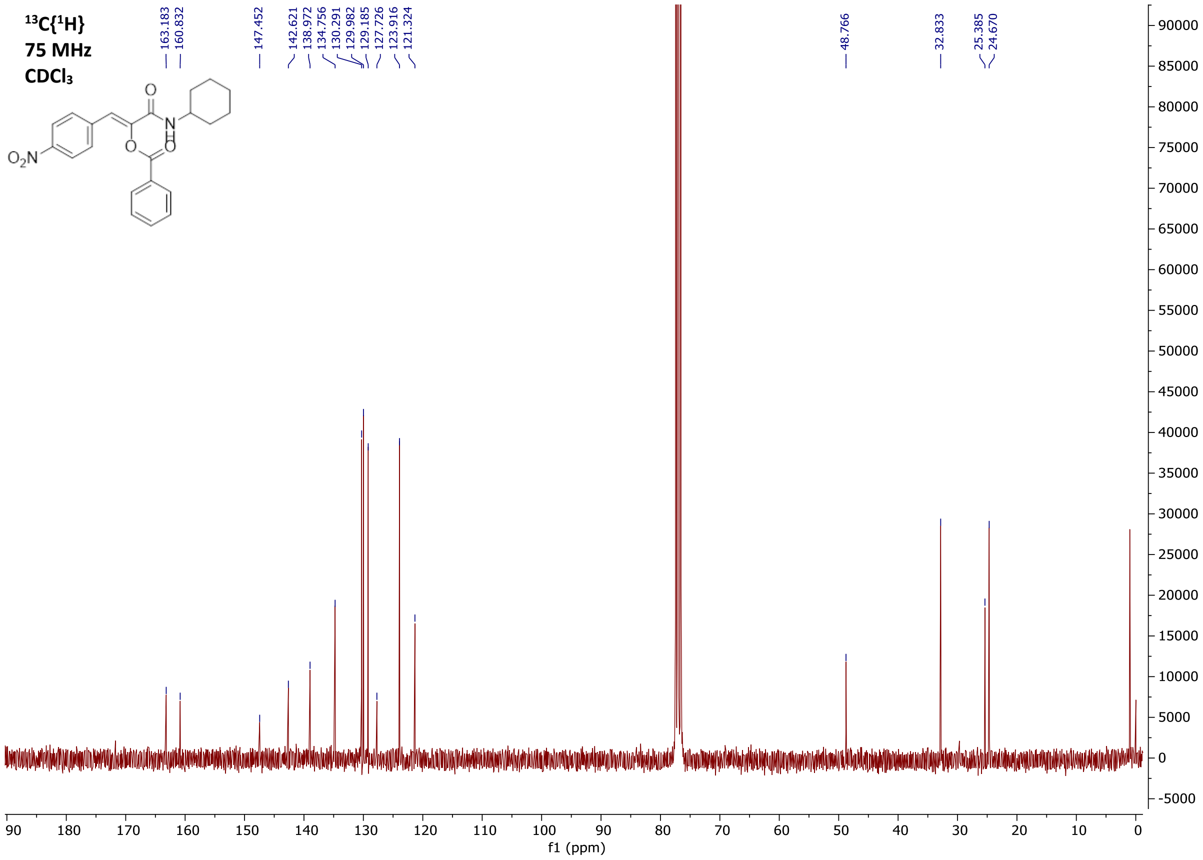

Compound **2e**

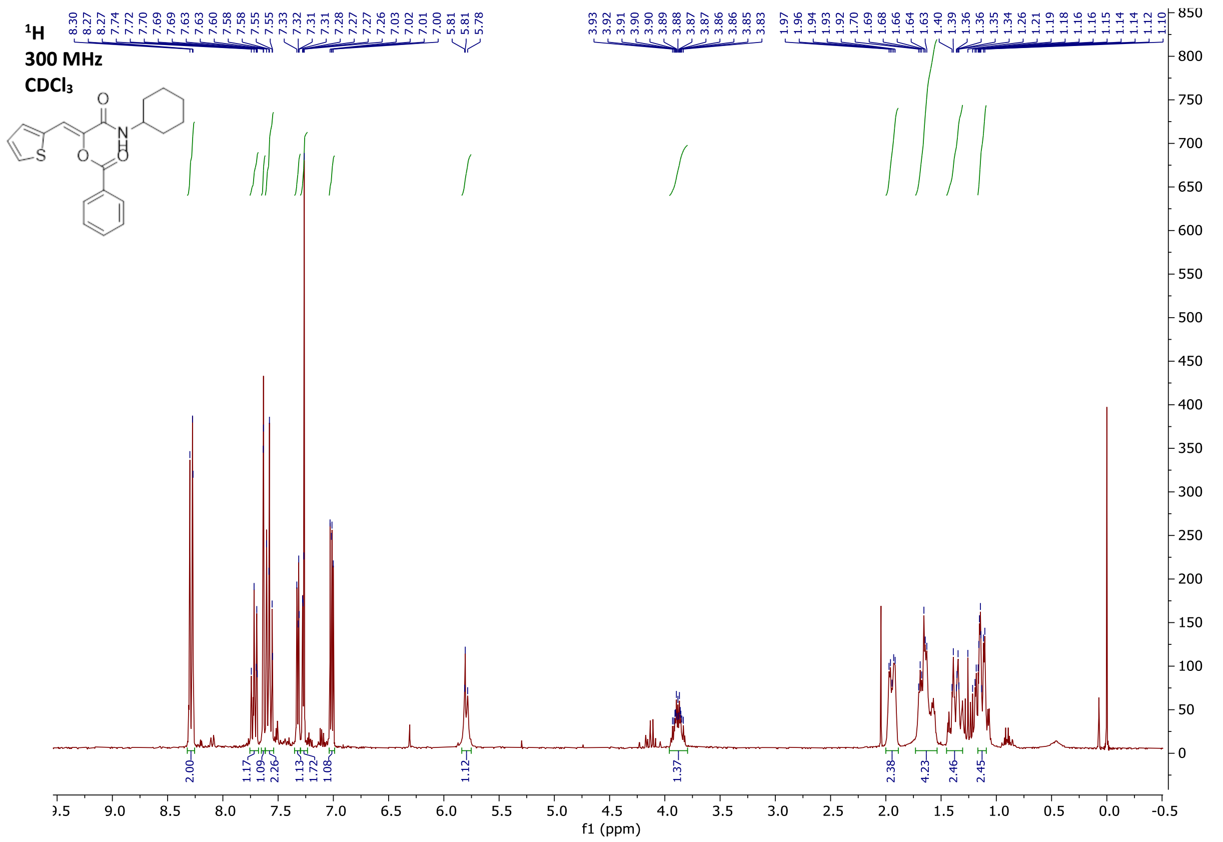

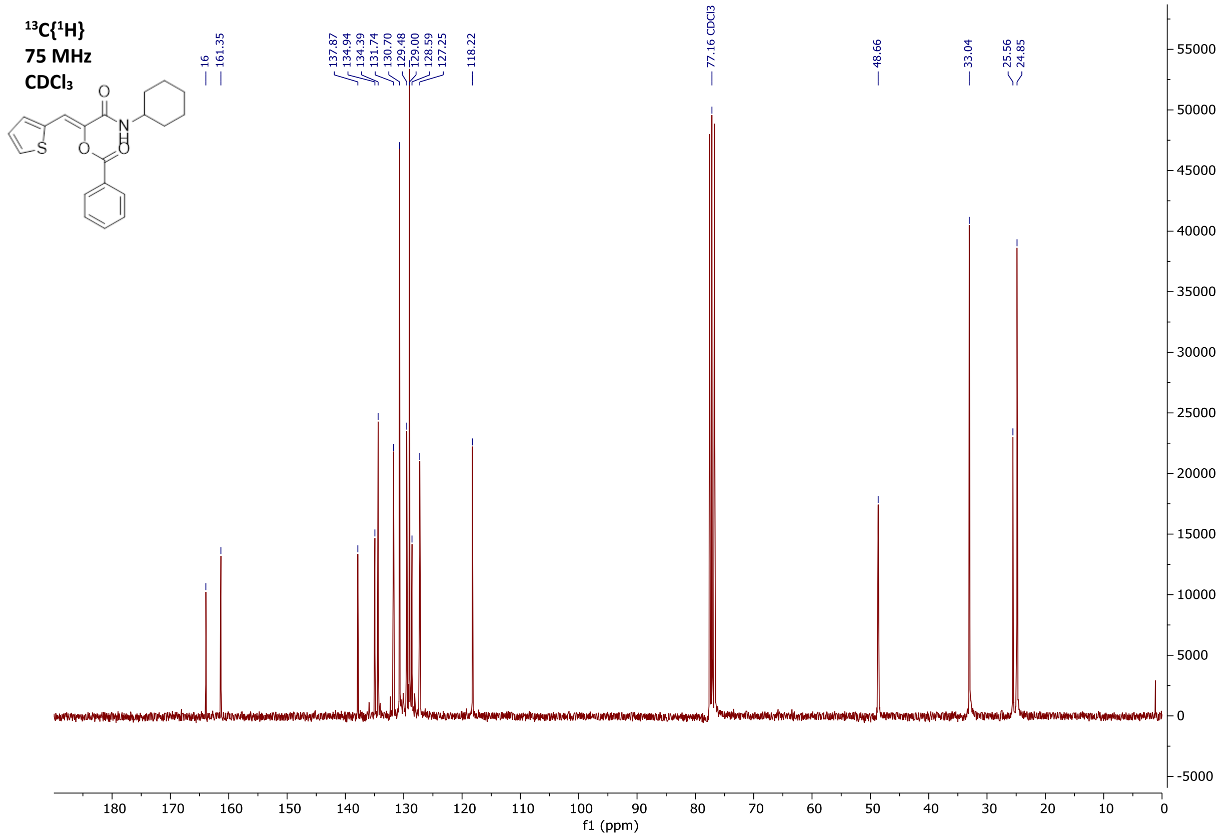

Compound **2f**

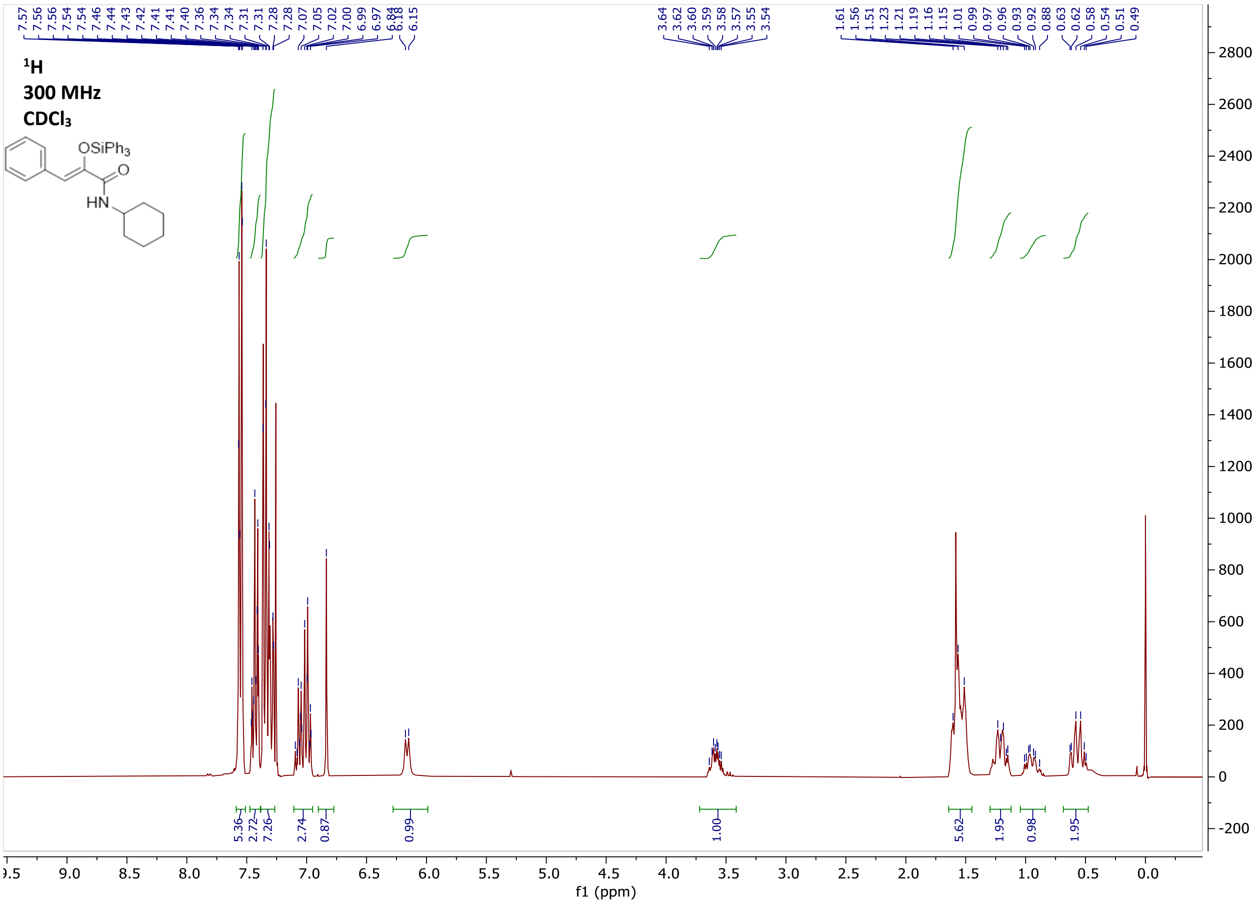

^{1}H
300 MHz
CDCl$_3$
OSiPh$_3$
O
HN
7.57 7.56 7.56 7.54 7.54 7.46 7.44 7.43 7.42 7.41 7.41 7.40 7.36 7.34 7.34 7.31 7.31 7.28 7.28 7.07 7.05 7.02 7.00 6.99 6.97 6.84 6.18 6.15
3.64 3.62 3.60 3.59 3.58 3.57 3.55 3.54
1.61 1.56 1.51 1.23 1.21 1.19 1.16 1.15 1.01 0.99 0.97 0.96 0.93 0.92 0.88 0.63 0.62 0.58 0.54 0.51 0.49
5.36 2.72 7.26 2.74 0.87 0.99 1.00 5.62 1.95 0.98 1.95
f1 (ppm)

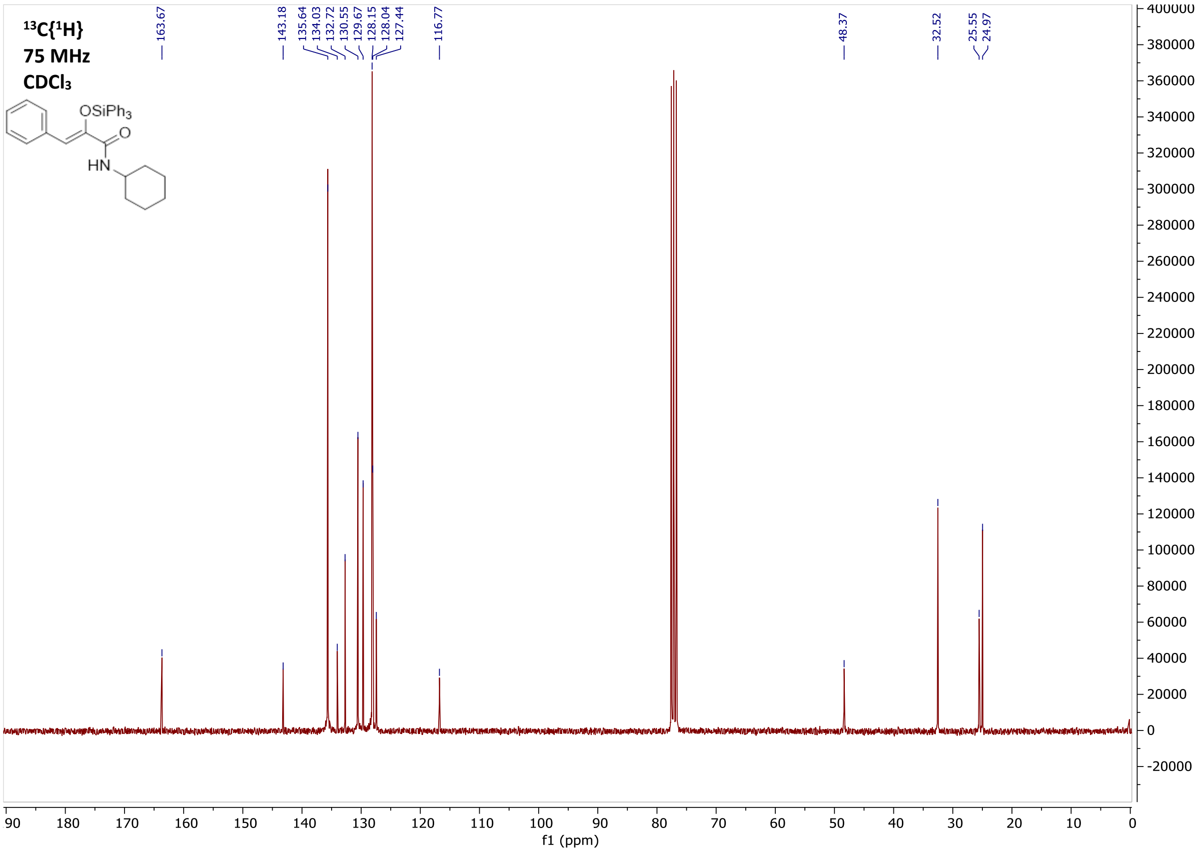

^{13}C{^{1}H}
75 MHz
CDCl$_3$
OSiPh$_3$
O
HN
163.67 143.18 135.64 134.03 132.72 130.55 129.67 128.15 128.04 127.44 116.77 48.37 32.52 25.55 24.97
f1 (ppm)

Compound **2g**

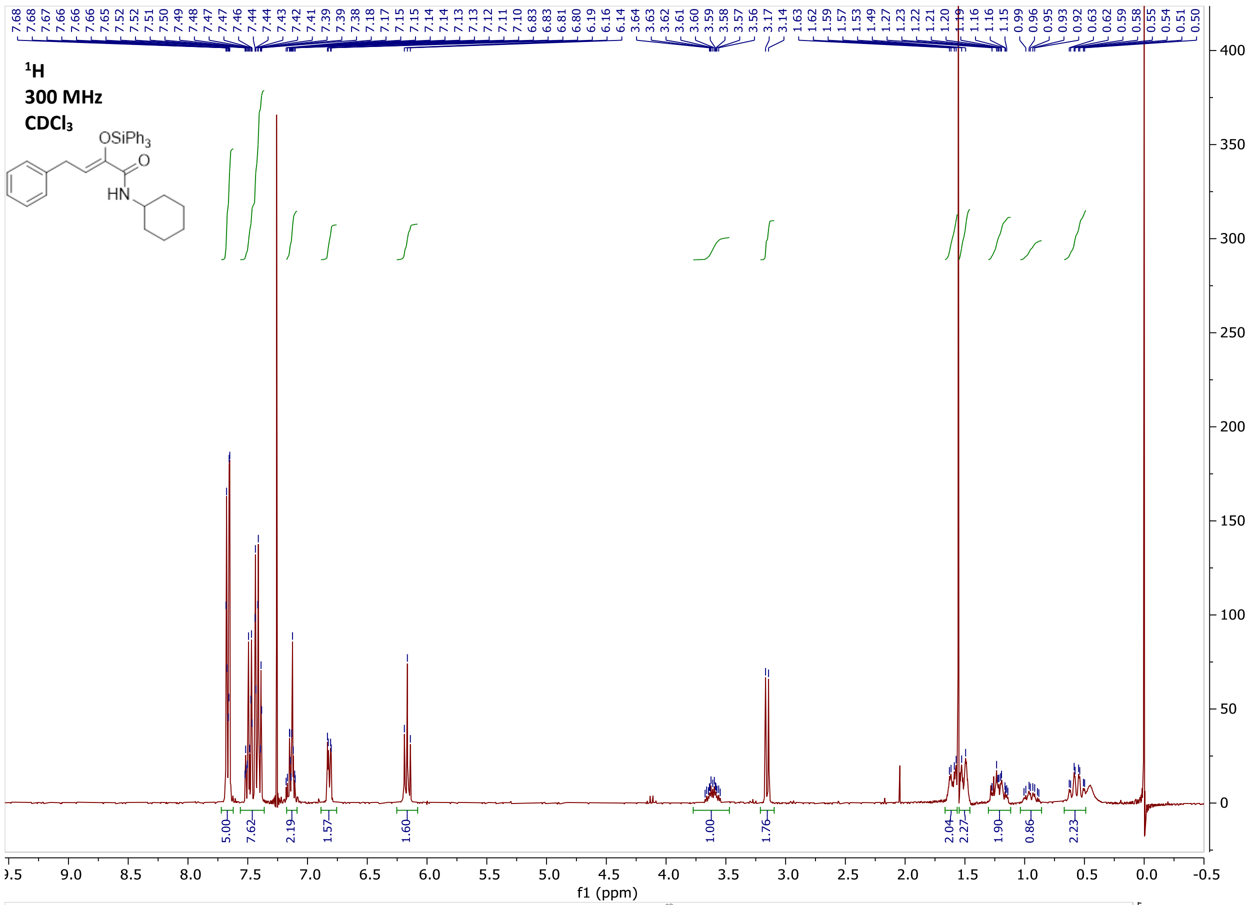

^{1}H
300 MHz
$CDCl_3$
OSiPh3
O
HN

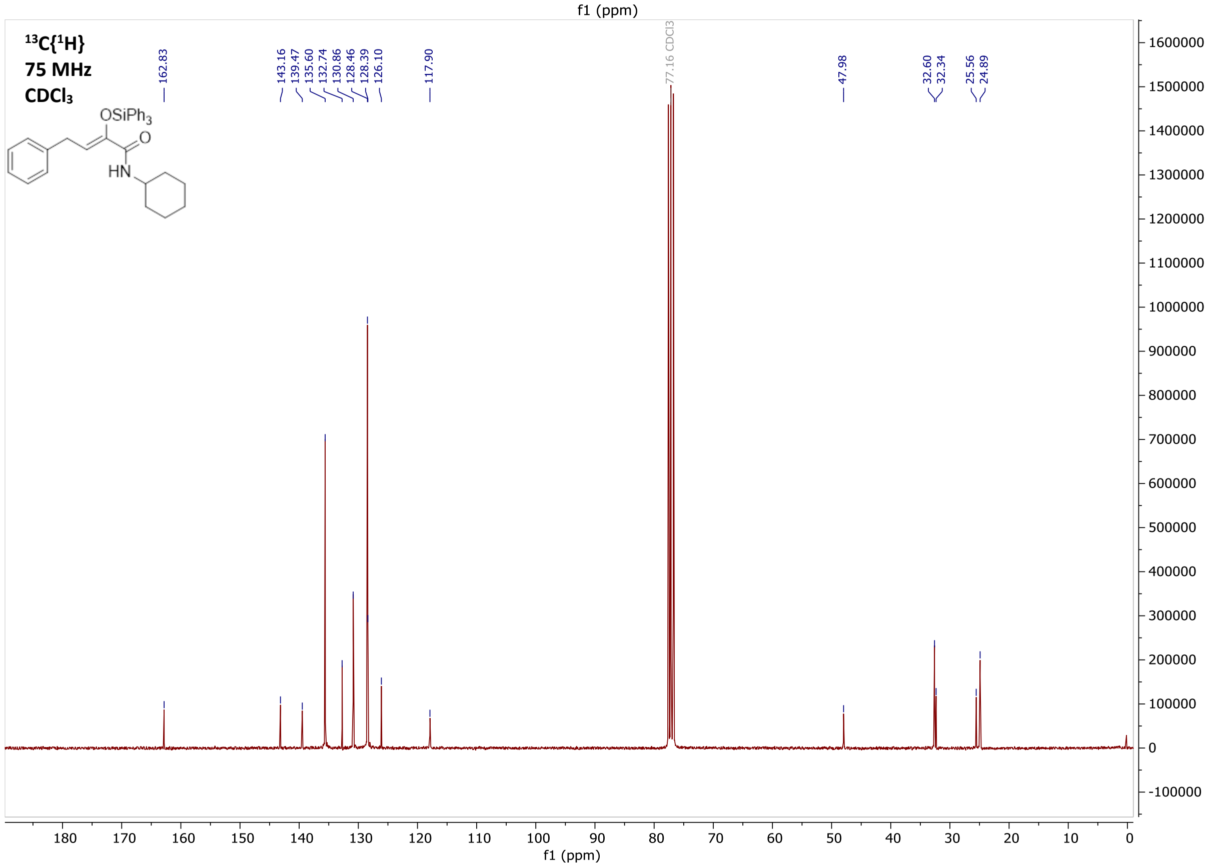

$^{13}C\{^{1}H\}$
75 MHz
$CDCl_3$
162.83
143.16
139.47
135.60
132.74
130.86
128.46
128.39
126.10
117.90
77.16 CDCl3
47.98
32.60
32.34
25.56
24.89
f1 (ppm)

Compound **2h**

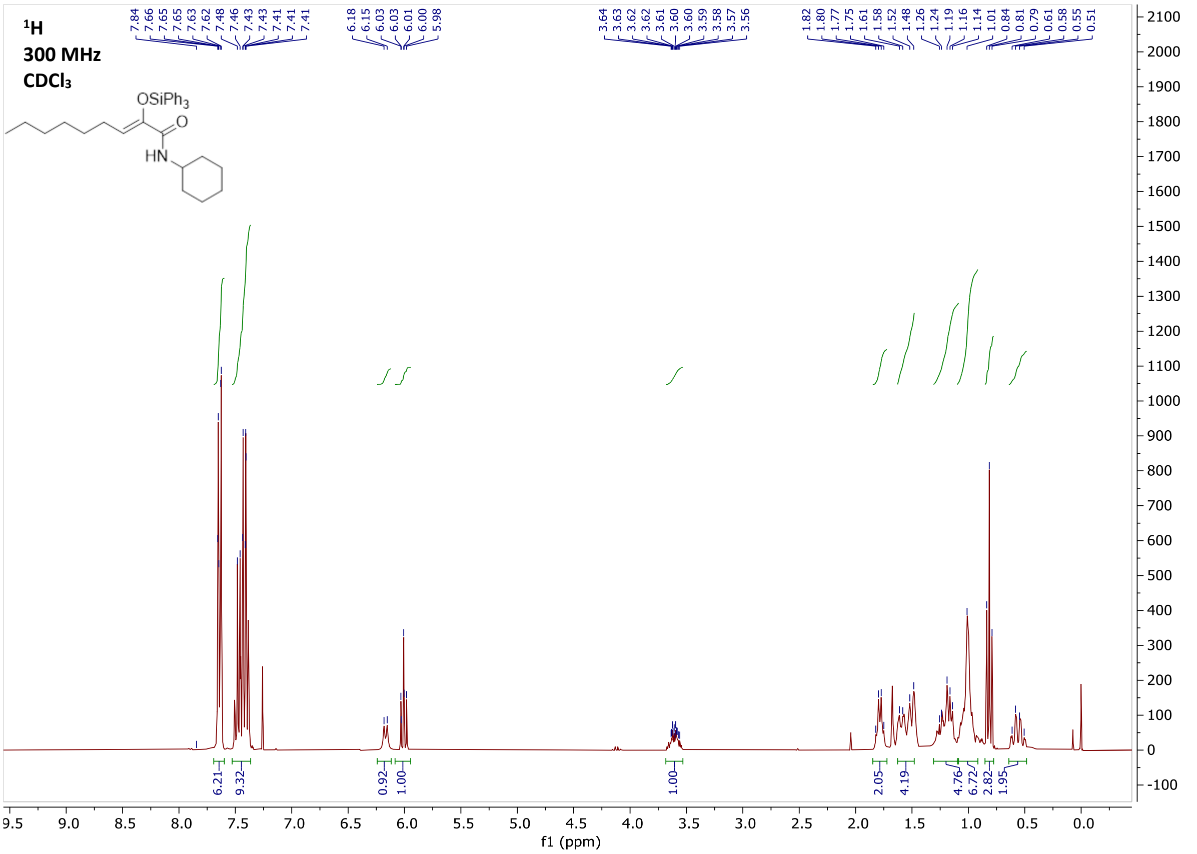

¹H
300 MHz
CDCl₃
OSiPh₃
O
HN
7.84 7.66 7.65 7.65 7.63 7.62 7.48 7.46 7.43 7.43 7.43 7.41 7.41 7.41
6.18 6.15 6.03 6.03 6.01 6.00 5.98
3.64 3.63 3.62 3.62 3.61 3.60 3.60 3.59 3.58 3.57 3.56
1.82 1.80 1.77 1.75 1.61 1.58 1.52 1.48 1.26 1.24 1.19 1.16 1.14 1.01 0.84 0.81 0.79 0.61 0.58 0.55 0.51
6.21 9.32 0.92 1.00 1.00 2.05 4.19 4.76 6.72 2.82 1.95
f1 (ppm)

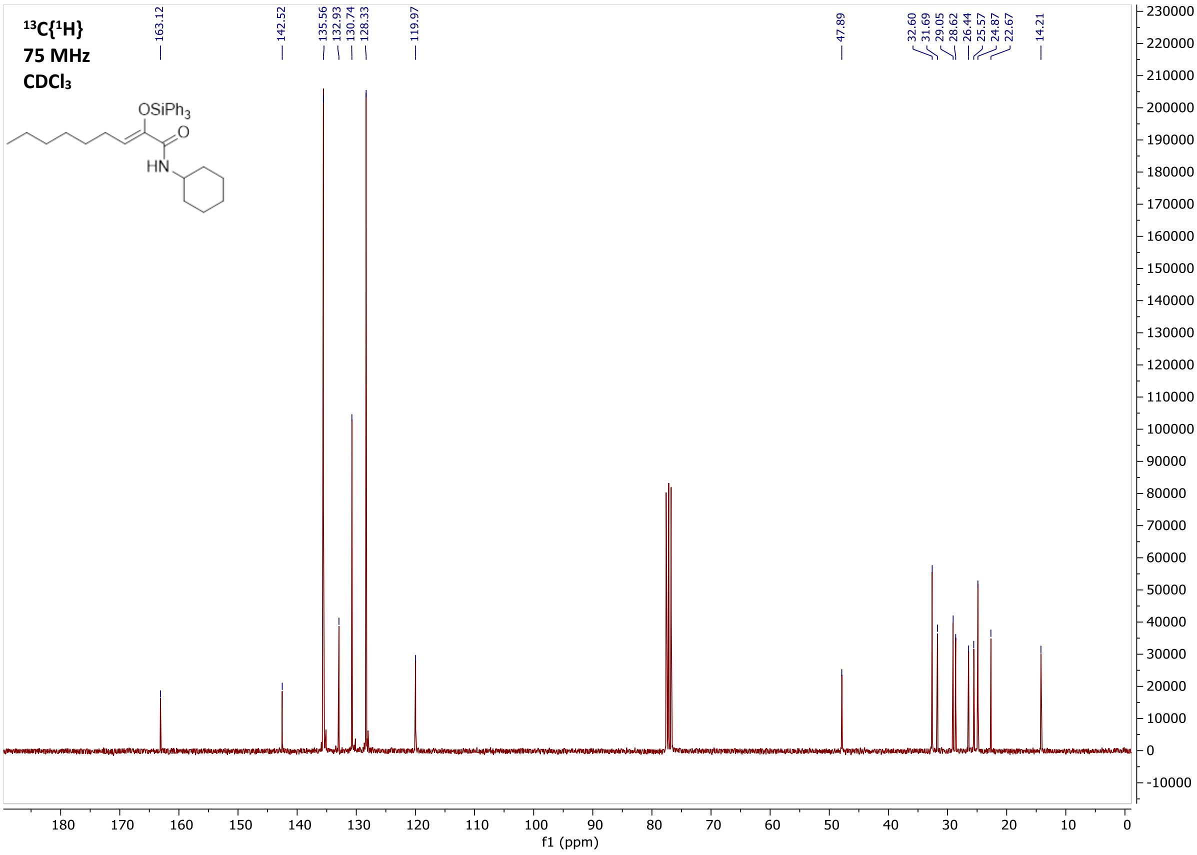

¹³C{¹H}
75 MHz
CDCl₃
OSiPh₃
O
HN
163.12 142.52 135.56 132.93 130.74 128.33 119.97 47.89 32.60 31.69 29.05 28.62 26.44 25.57 24.87 22.67 14.21
f1 (ppm)

Compound **2i**

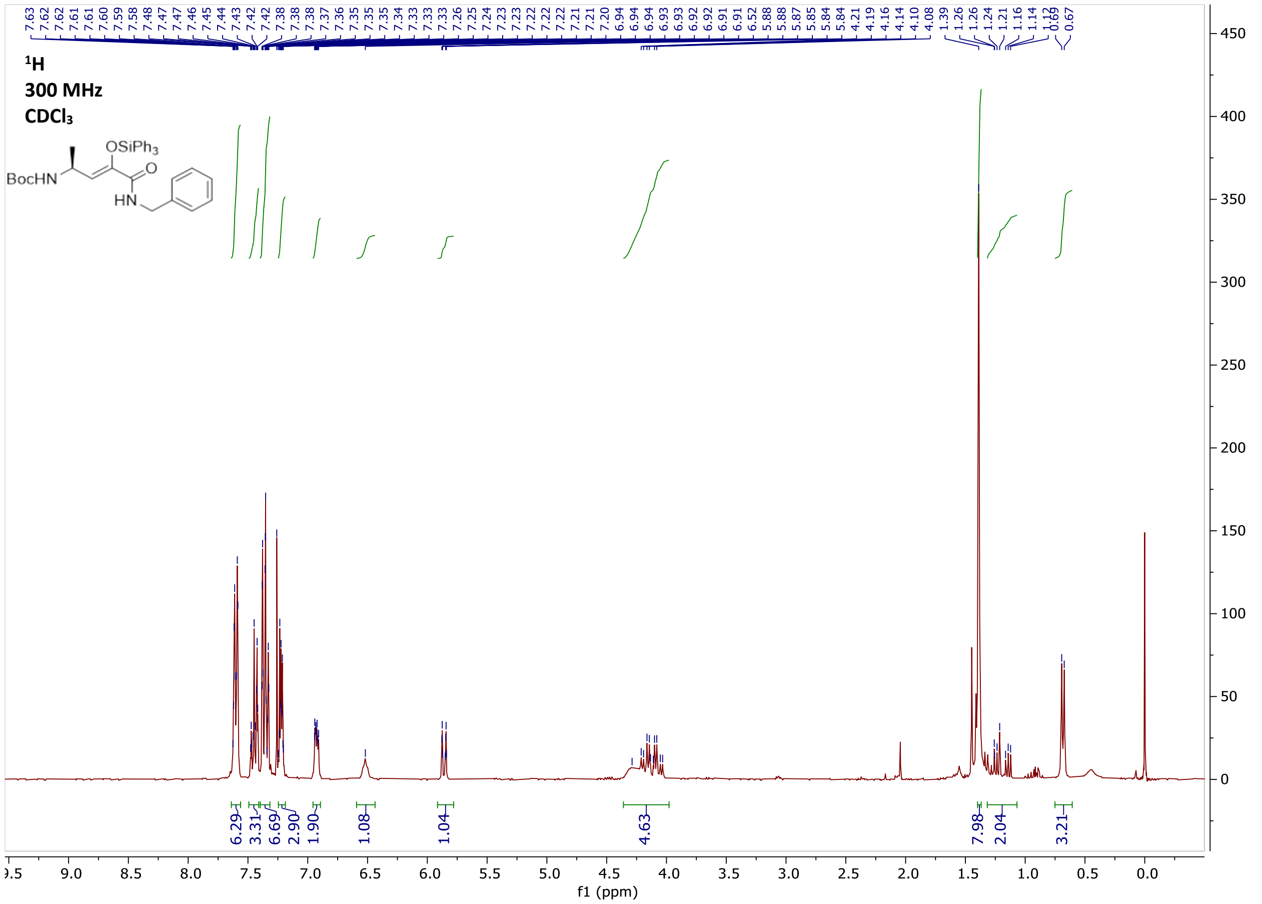

¹H
300 MHz
CDCl₃
OSiPh₃
BocHN
O
HN
f1 (ppm)

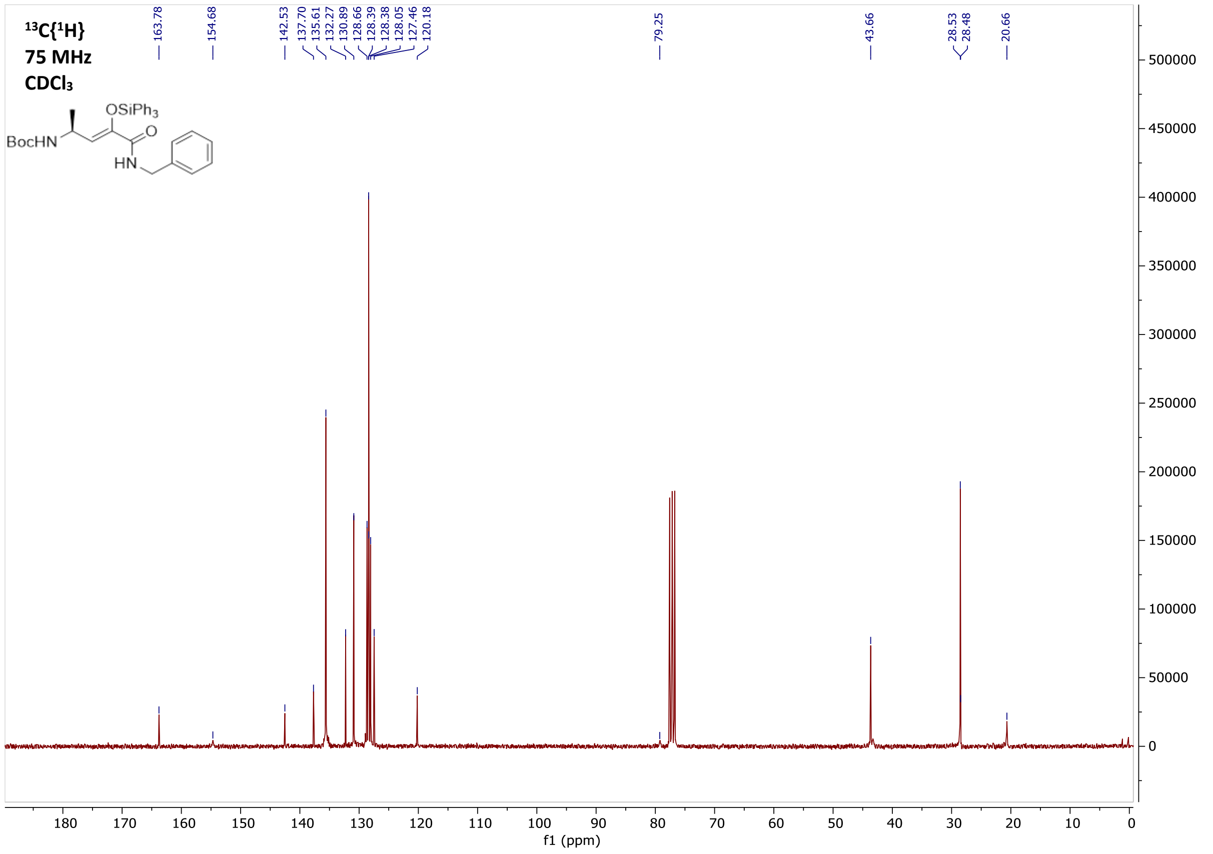

¹³C{¹H}
75 MHz
CDCl₃
OSiPh₃
BocHN
O
HN
f1 (ppm)

Compound **2l**

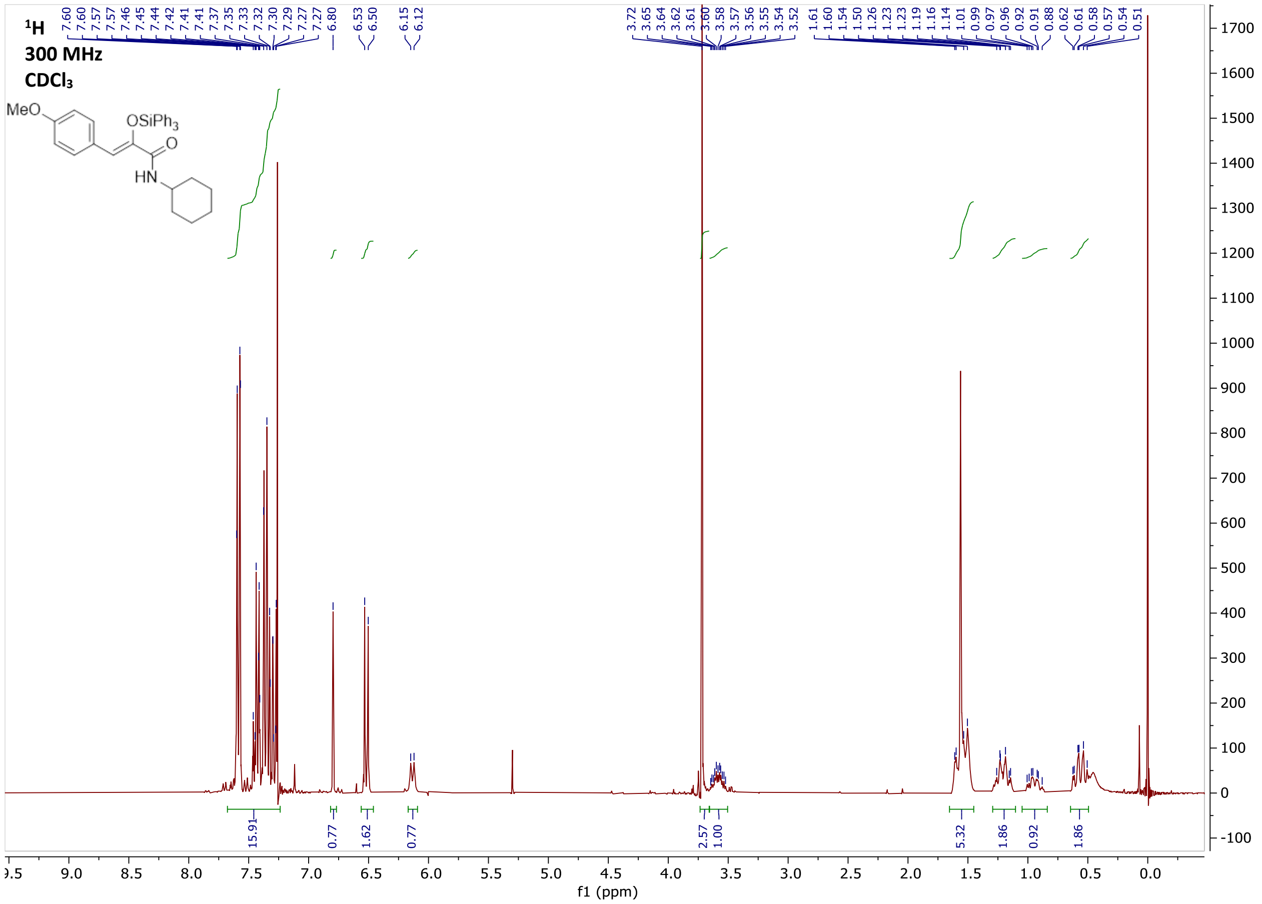

1H
300 MHz
CDCl3
MeO
OSiPh3
O
HN
7.60 7.60 7.57 7.57 7.46 7.45 7.44 7.42 7.41 7.41 7.37 7.35 7.33 7.32 7.30 7.29 7.27 7.27 6.80 6.53 6.50 6.15 6.12
3.72 3.65 3.64 3.62 3.61 3.60 3.58 3.57 3.56 3.55 3.54 3.52 1.61 1.60 1.54 1.50 1.26 1.23 1.23 1.19 1.16 1.14 1.01 0.99 0.97 0.96 0.92 0.91 0.88 0.62 0.61 0.58 0.57 0.54 0.51
15.91 0.77 1.62 0.77 2.57 1.00 5.32 1.86 0.92 1.86
f1 (ppm)

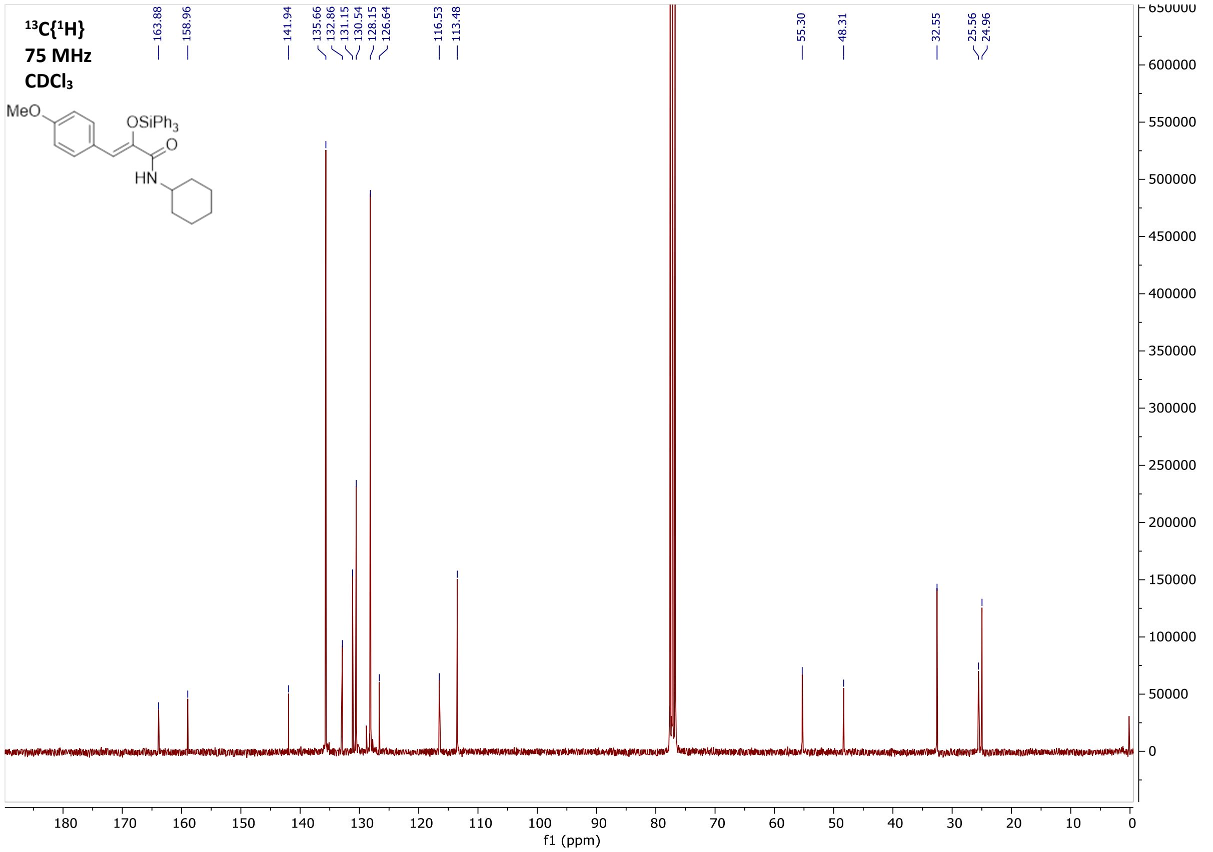

13C{1H}
75 MHz
CDCl3
MeO
OSiPh3
O
HN
163.88 158.96 141.94 135.66 132.86 131.15 130.54 128.15 126.64 116.53 113.48 55.30 48.31 32.55 25.56 24.96
f1 (ppm)

Compound **3**

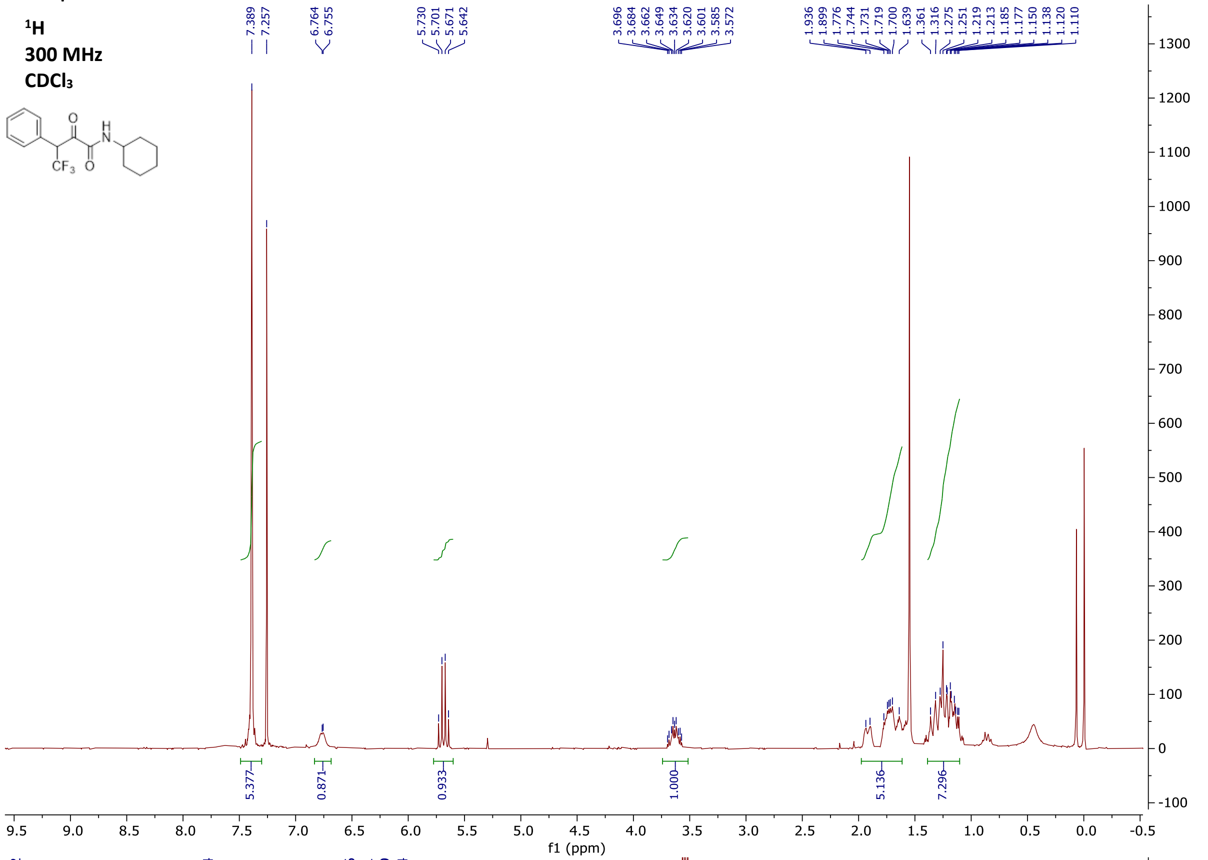

¹H
300 MHz
CDCl₃
7.389
7.257
6.764
6.755
5.730
5.701
5.671
5.642
3.696
3.684
3.662
3.649
3.634
3.620
3.601
3.585
3.572
1.936
1.899
1.776
1.744
1.731
1.719
1.700
1.639
1.361
1.316
1.275
1.251
1.219
1.213
1.185
1.177
1.150
1.138
1.120
1.110
5.377
0.871
0.933
1.000
5.136
7.296
f1 (ppm)

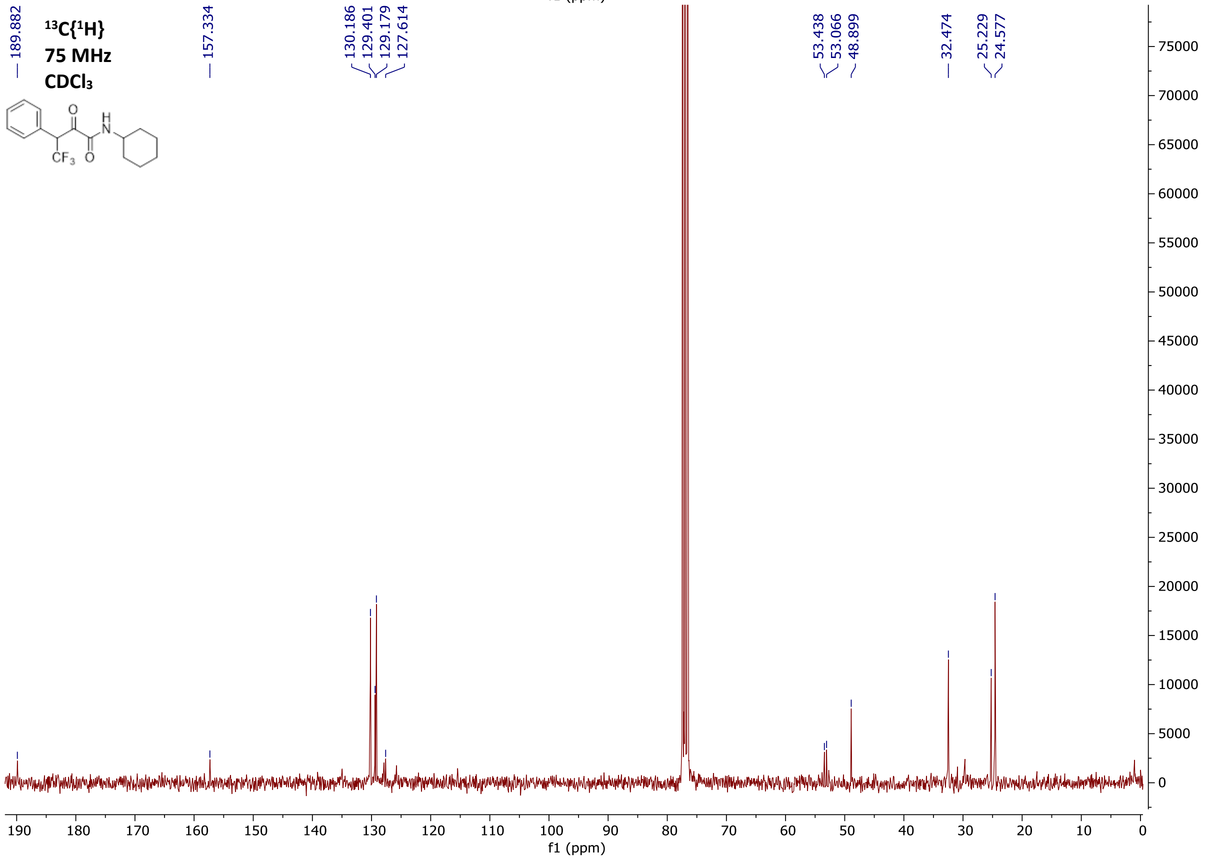

¹³C{¹H}
75 MHz
CDCl₃
189.882
157.334
130.186
129.401
129.179
127.614
53.438
53.066
48.899
32.474
25.229
24.577
f1 (ppm)

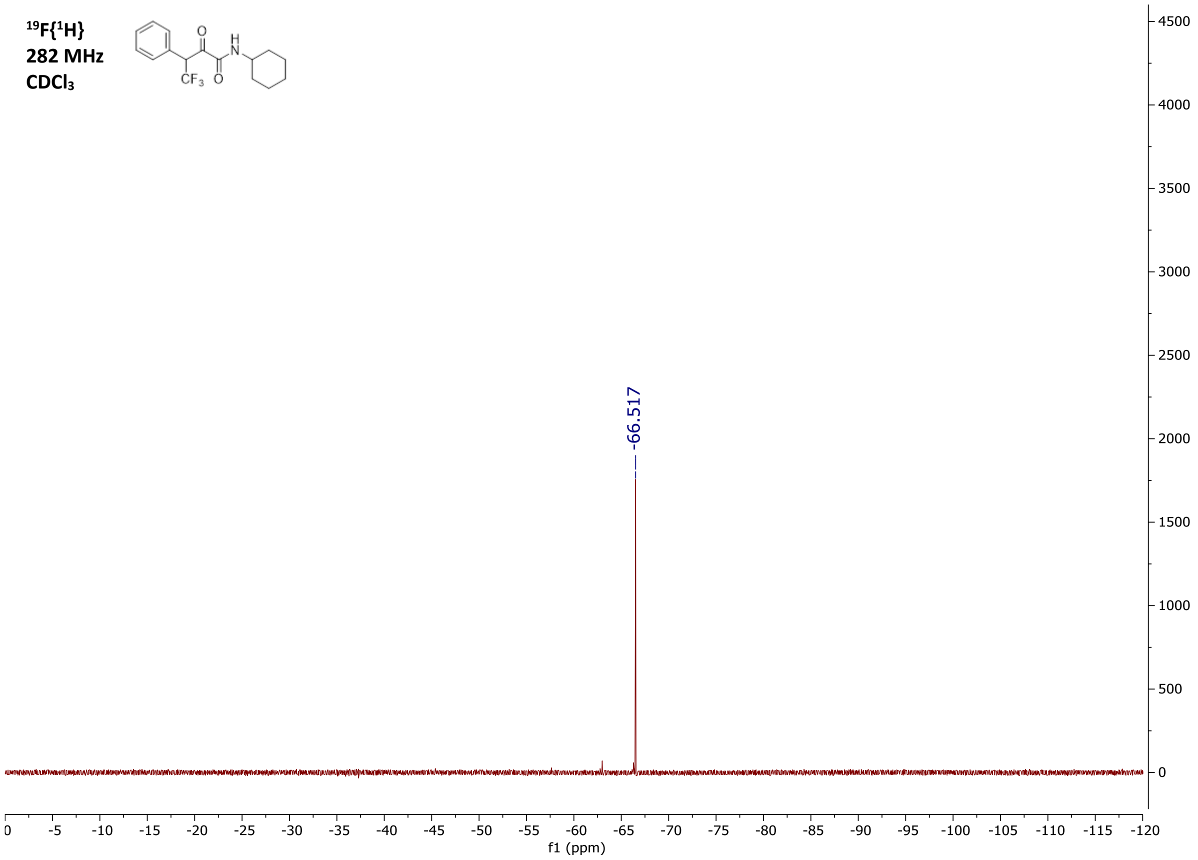

19F{1H}
282 MHz
CDCl3
-66.517
f1 (ppm)

Compound **4**

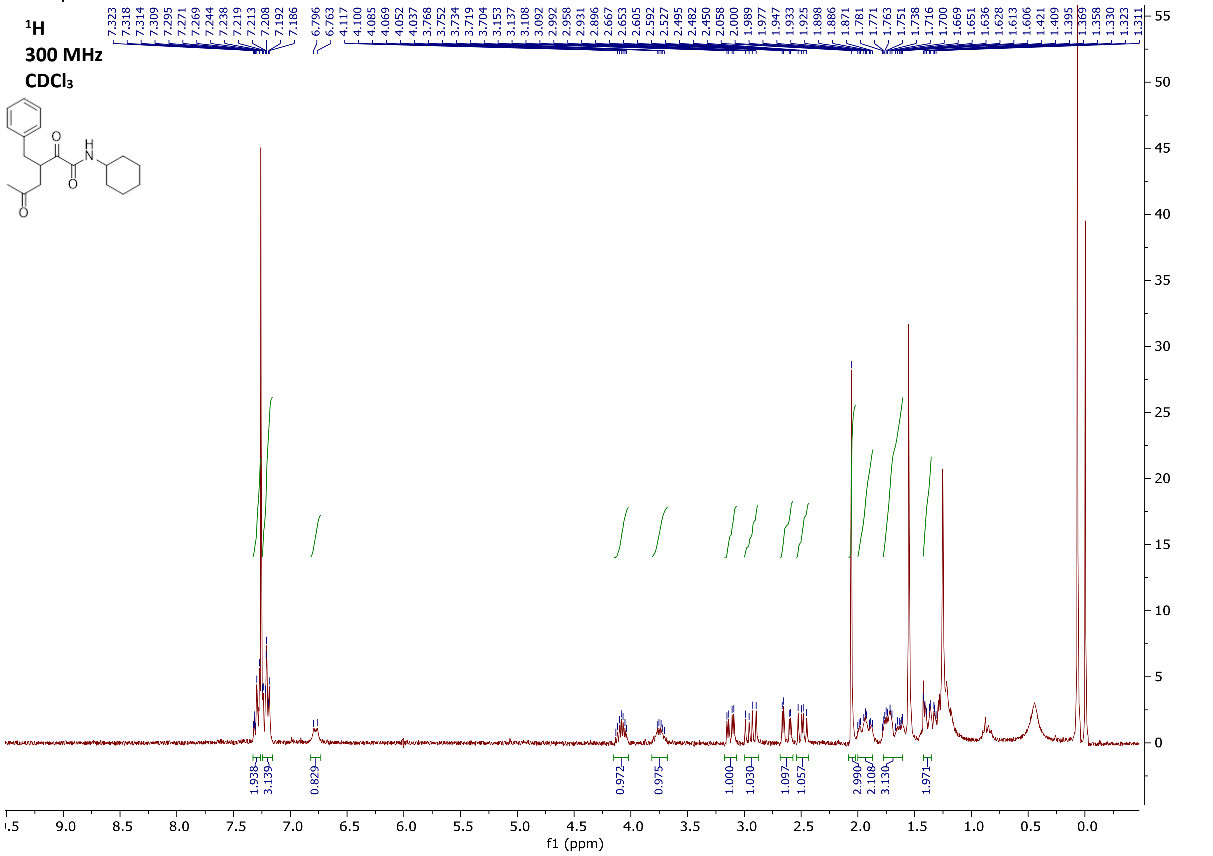

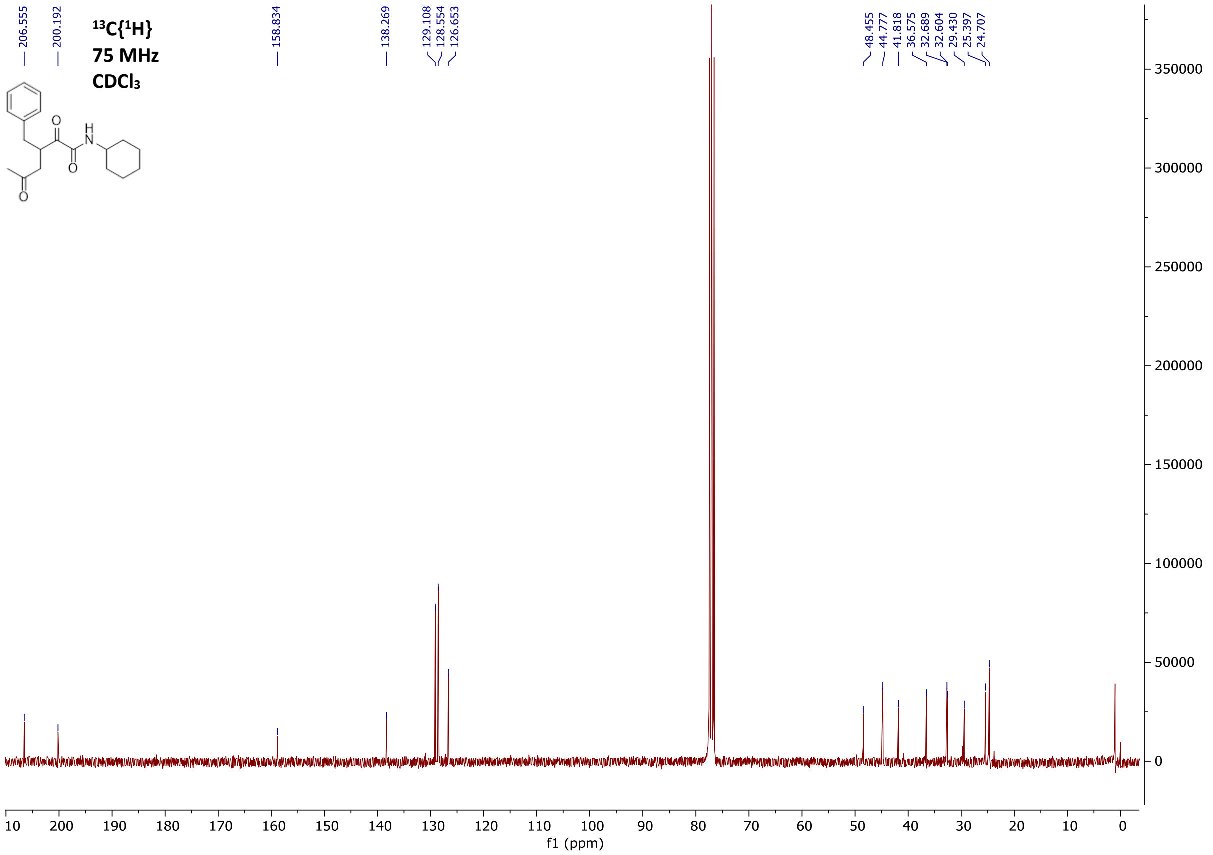

Supplement: Supplementary file 1 — jo1c00278_si_001.pdf [file jo1c00278_si_001.pdf]
